# Efficacy and safety of non-pharmacological therapies for primary insomnia: a network meta-analysis

**DOI:** 10.3389/fneur.2025.1607903

**Published:** 2025-07-29

**Authors:** Qin-hong Zhang, Yong-jian Liu, Guanhu Yang, Mei-yi Zhu, Jia-hui Yang, Lun Li, Xiu-mei Yan, Qi-lin Liu, Jin-huan Yue, Xiao-ling Li, Yi-ming Li, Tian-cheng Xu, Fan Jiang

**Affiliations:** ^1^Shenzhen Frontiers in Chinese Medicine Research Co., Ltd., Shenzhen, China; ^2^Heilongjiang University of Chinese Medicine, Harbin, China; ^3^Jiangxi University of Chinese Medicine, Nanchang, China; ^4^Research Department, Swiss TCM University, Bad Zurzach, Switzerland; ^5^Faculty of Chinese Medicine, Macau University of Science and Technology, Macau, China; ^6^Department of Specialty Medicine, Ohio University, Athens, OH, United States; ^7^Division of CT and MRI, First Affiliated Hospital of Heilongjiang University of Chinese Medicine, Harbin, China; ^8^Key Laboratory of Acupuncture and Medicine Research of Ministry of Education, Nanjing University of Chinese Medicine, Nanjing, China; ^9^The Affiliated Hospital of Jiangxi University of Chinese Medicine, Nanchang, China

**Keywords:** primary insomnia, cognitive behavioral therapy, acupuncture, acupressure, cupping therapy

## Abstract

**Background:**

Primary insomnia (PI) is a prevalent sleep disorder that significantly impacts quality of life. While pharmacological treatments are common, concerns about side effects and dependency have led to increased interest in non-pharmacological alternatives. This study systematically evaluates the efficacy and safety of various non-pharmacological therapies for adult PI through a network meta-analysis, providing evidence-based guidance for clinicians.

**Methods:**

We analyzed 53 randomized controlled trials (RCTs) involving 4,181 adults with PI. The included studies assessed 11 non-pharmacological interventions, such as acupuncture, acupressure, cupping therapy, and cognitive behavioral therapy (CBT), alongside control groups (e.g., placebo, waitlist, and pharmacological comparators). Primary outcomes included the Pittsburgh Sleep Quality Index (PSQI), total sleep time (TST), sleep efficiency (SE), and sleep latency (SL). Data synthesis was performed using STATA 17 software with a random-effects model, and evidence quality was appraised using the GRADE framework.

**Results:**

Pooled analyses revealed that all seven non-pharmacological therapies significantly improved PI outcomes compared to controls. Acupuncture reduced PSQI scores by −2.71 points (95% confidence interval (CI): −4.94 to −0.49) versus waitlist, while acupuncture showed a − 1.81 point reduction (95% CI: −2.93 to −0.68). For SE, acupressure and CBT increased SE by 1.48% (95% CI: 0.56–2.39) and 1.34% (95% CI: 0.70–1.98), respectively, compared to SH. Notably, CBT and acupressure shortened SL by approximately 10 min (e.g., CBT: −10.15 min, 95% CI: −11.79 to −8.52 vs. benzodiazepines), while acupressure extended TST by 2.07 h (95% CI: 0.46–3.68). SUCRA rankings identified CBT as the most effective for reducing SL (85.8% probability) and improving SE (89.2%), whereas acupuncture excelled in increasing TST (84.8%). Adverse events were infrequent and mild, primarily limited to transient localized reactions in acupuncture studies.

**Conclusion:**

This study demonstrates that non-pharmacological therapies are effective and safe in managing PI, with CBT, acupuncture, and acupressure emerging as optimal choices for specific sleep parameters. These findings advocate non-pharmacological interventions into clinical practice and offer clinicians valuable insights for selecting appropriate treatment modalities for PI management. However, study limitations like heterogeneity and small sample sizes highlight the need for larger, well-designed RCTs. Future studies should use standardized measures for more specific insomnia assessment.

## 1 Introduction

Primary insomnia (PI) is a common sleep disorder characterized by difficulty falling asleep, maintaining sleep, or early morning awakenings, occurring at least three times per week for a minimum of 3 months. These symptoms are frequently accompanied by clinical significant distress and daytime functional impairments, including irritability, fatigue, and decreased productivity. With a global prevalence reaching 22.1% (1), PI substantially compromises health-related quality of life worldwide (2). Although earlier guidelines recommended pharmacological like benzodiazepine (3, 4), their long-term use is limited by adverse effects such as memory impairment, psychological disturbances, and elevated risk of depression/anxiety. Consequently, non-pharmacological interventions like cognitive behavioral therapy (CBT) were historically regarded as adjunctive options.

Recent paradigm shifts are evident in updated guidelines. The European Sleep Research Society now designates non-pharmacological therapies (particularly CBT) as first-line PI treatments, reserving short-term pharmacotherapy only for refractory cases (5). This reflects growing recognition of non-drug approaches as primary management strategies.

Among these interventions, CBT demonstrates robust efficacy in improving psychological outcomes and sleep efficiency (6). Acupuncture, another evidence-based modalities, exerts therapeutic effects through neuroendocrine modulation [e.g., melatonin regulation (7), and autonomic nervous system regulation sympathetic suppression/parasympathetic enhancement (8)]. Clinical study confirms acupuncture’s particular effectiveness in prolonging sleep duration (9). Notably, insomnia severity positively correlates with patients’ pursuit of physical therapies (10). However, existing randomized controlled trials (RCTs) on non-pharmacological treatments frequently exhibit methodological limitations—including small samples and placebo effects—compromising conclusions about their efficacy and safety profiles.

Epidemiological trends reveal increasing PI prevalence among younger populations (11). Adolescents, for instance, average merely 6.25 h of weekday sleep, with 65% experiencing >30-min sleep latency (SL). Despite this demographic shift, most meta-analyses disproportionately focus on elderly cohorts, neglecting younger age groups (12).

To address these gaps, this study conducts a network meta-analysis of RCTs evaluating non-pharmacological PI therapies. By systematically comparing efficacy and safety of various non-pharmacological treatments in adult, this study aims to establish evidence-based clinical recommendations for optimal therapeutic selection.

## 2 Materials and methods

### 2.1 Literature search

Two researchers independently performed systematic searches across eight databases (China National Knowledge Infrastructure, VIP, Wanfang, SinoMed, PubMed, EMBASE, Cochrane Library, and Web of Science) from inception through December 24, 2023. The search strategy combined subject terms and free-text keywords using the following terms: (primary insomnia) AND (acupuncture OR electroacupuncture OR acupressure OR cupping OR physical therapy OR cognitive behavioral therapy) AND (randomized controlled trial). The detailed search strategy is presented in Supplementary Tables S1–S8. Manual searches of reference lists from eligible RCTs and meta-analyses supplemented electronic retrieval.

### 2.2 Inclusion criteria

(1) Study design: Peer-reviewed RCTs published in Chinese or English.
(2) Participants: Adults (≥18 years) meeting DSM-IV-TR criteria (American Psychiatric Association) for PI (13), supplemented by the Chinese guidelines for adult insomnia diagnosis (14). Although DSM-5 represents the current standard, we retained DSM-IV-TR criteria because: (a) 77% of included trials used this framework; (b) core PI diagnostic features remained consistent across editions; and (c) this maintained methodological alignment with most included studies. ICSD-3 criteria (American Academy of Sleep Medicine, 2014) were additionally extracted when reported.
(3) Interventions: Eleven comparators were evaluate: acupuncture, acupressure, cupping therapy (CUP), CBT, sleep hygiene (SH), repetitive transcranial magnetic stimulation (rTMS), relaxation therapy (RT), placebo (sham acupuncture or sham treatment) (PLA), nenzodiazepines (BZD), non-benzodiazepines (NBZD), and waiting list control (Wait List).
(4) Outcome measures: Primary outcomes included Pittsburgh Sleep Quality Index (PSQI), total sleep time (TST), sleep efficiency (SE), and sleep latency (SL), selected based on: (1) consistent reporting in most included studies, and (2) validated psychometric properties in insomnia research. The PSQI is a well-validated self-report questionnaire designed to assess sleep quality over a one-month period, comprising seven components (each scored 0–3; total range: 0–21), with higher scores indicating poorer sleep quality (15). Key sleep parameters, including TST, SE, and SL, were derived from objective measures—either polysomnography (the gold standard for sleep assessment) or actigraphy (a wearable-based method estimating sleep parameters via movement detection)—where available. In cases where objective data were lacking, validated sleep diaries served as an alternative for self-reported TST, SE, and SL. SE was defined as the ratio of actual sleep duration to time spent in bed, while SL represented the duration from lights-out to sleep onset, and TST referred to the total sleep duration per night from sleep initiation to awakening. Although wake after sleep onset (WASO) and early morning awakening are clinically relevant to insomnia, these metrics were excluded from the primary analysis due to inconsistent reporting (available in fewer than 40% of studies) and significant methodological variability across trials.

While the PSQI encompasses a broad range of sleep-related domains—including environmental disturbances and physical symptoms (e.g., nocturnal breathing irregularities)—it remains a widely accepted tool in insomnia research, demonstrating strong correlations with insomnia severity. However, to improve diagnostic specificity, future studies may consider supplementing the PSQI with the Insomnia Severity Index (ISI), which specifically targets core insomnia symptoms, such as difficulties in sleep initiation, maintenance, and associated daytime impairment, thereby enhancing clinical sensitivity and precision in outcome assessment.

### 2.3 Exclusion criteria

(1) Study types: Excluded studies included case reports, retrospective studies, animal experiments, reviews, cell experiments, and experience summaries.
(2) Participants: Studies were excluded if (a) the diagnostic criteria were absent or unclear, or (b) the participants had concomitant psychiatric disorders or other organic diseases.
(3) Interventions: Studies comparing known effective treatments with experimental therapies were excluded.
(4) Outcome measures: Studies were excluded if (a) they lacked outcome measures or (b) the outcome data could not be obtained, and the authors could not be contacted.

### 2.4 Literature screening and data extraction

Two researchers independently screened all retrieved records from electronic databases, including clinical trials and the references of relevant systematic reviews or meta-analyses, based on predefined eligibility criteria. Duplicate records and studies available only as abstracts were excluded. Disagreements between researchers were resolved through discussion or, if necessary, by consultation with a third independent researcher.

### 2.5 Risk of Bias assessment

To assess methodological quality, two researchers independently evaluated the included studies using the Cochrane Risk of Bias (ROB) 2.0 tool (16). This updated version addresses limitations in assessing study design and baseline group comparability by refining evaluation criteria and eliminating ambiguous terminology. ROB 2.0 examines multiple bias domains, including randomization, allocation concealment, baseline imbalances, deviations from intended interventions, missing data, outcome measurement, and selective reporting. Each bias is rated as “low,” “high,” or “unclear.” If disagreements occurred, a third researcher would be consulted to make the final decision.

### 2.6 Statistical analysis

All analyses were performed using STATA 17 software, with results visualized using relationship plots. For safety outcomes, random-effects models were applied to estimate relative risk (RR) with 95% confidence interval (CI). Treatment effects were quantified as mean differences (MD) or standardized MD, depending on data compatibility. Heterogeneity was evaluated using the *I*^2^ statistic, categorized as low (*I*^2^ < 30%), moderate (*I*^2^, 30–50%), or high (*I*^2^ > 50%). Sensitivity analyses were conducted to test result robustness.

For primary outcomes, a network meta-analysis was performed to map associations between interventions. Node sizes reflected participant numbers per intervention, while connecting line widths represented the number of trials comparing paired interventions. Direct and indirect evidence were synthesized to assess comparative effectiveness and safety, expressed as RR with 95% CI. Intervention rankings were derived using the Surface under the Cumulative Ranking (SUCRA) curve, where higher SUCRA values indicated superior efficacy. Ranking probabilities were computed cumulatively to generate a hierarchy of interventions. All tests were two-tailed, with statistical significance set at *p* < 0.05.

## 3 Results

### 3.1 Literature search results

**Figure 1:**
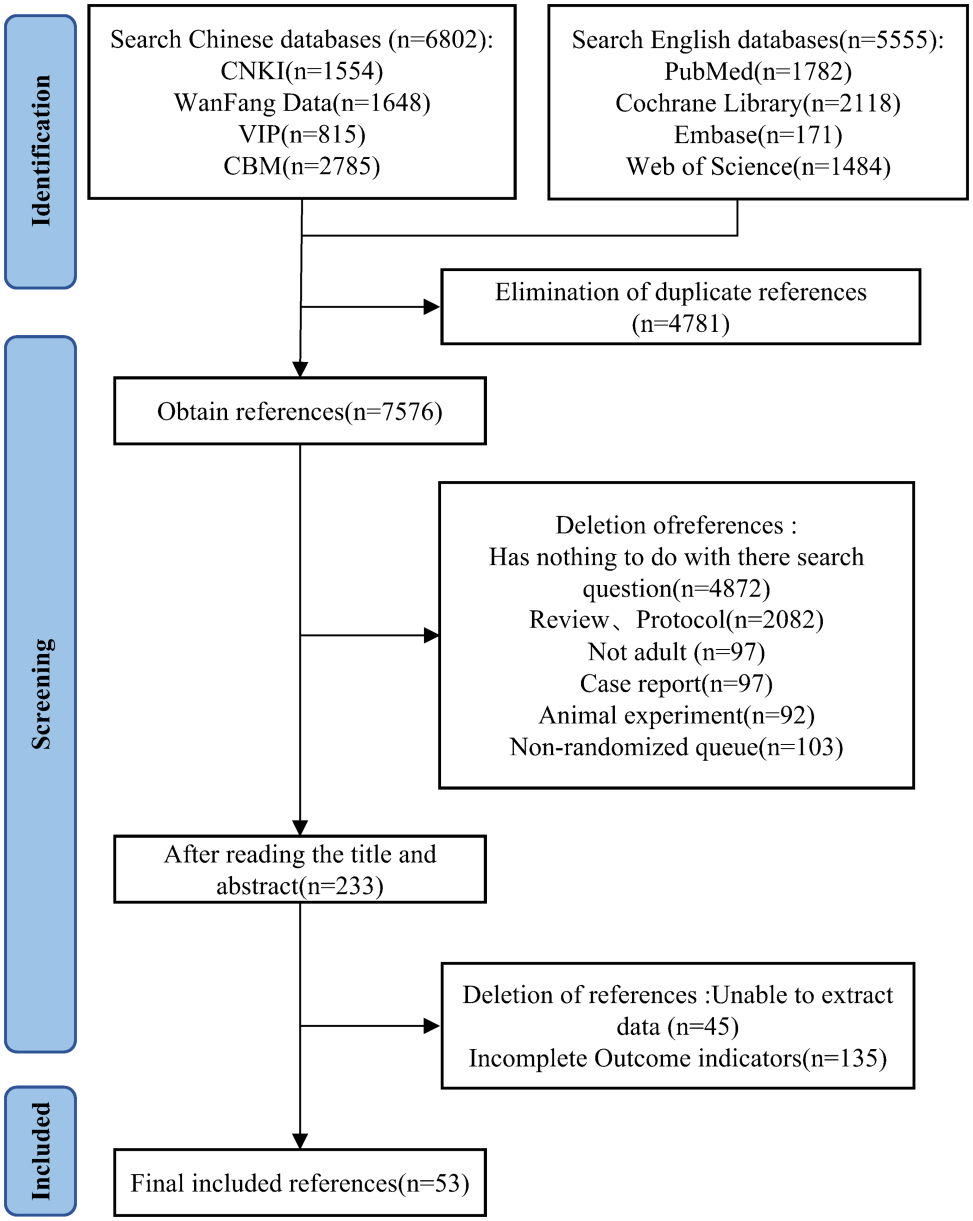
Flowchart of study selection.

A total of 12,357 studies were systematic retrieved during the literature search. After removing duplicates using Endnote 20, 7,576 records remained. By screening titles and abstracts, 7,343 articles were excluded. A further 180 articles were excluded after full-text evaluation, resulting in the final inclusion of 53 studies (17–69). The detailed literature search process is illustrated in Figure 1.

### 3.2 Basic characteristics of the included studies

The final analysis included 53 RCTs (total *N* = 4,181 participants), comprising 46 two-arm and 7 three-arm trials (17–69). These studies evaluated 11 interventions: acupuncture, acupressure, CBT, RT, CUP, SH, rTMS, BZD, NBZD, PLA, and Wait List. Detailed study characteristics are presented in Table 1.

**Table 1:** Characteristics of included studies.

Study	Sample size (T/C)	Age (year, mean ±SD; or range)	Gender, Female (%)	Treatment	Control	Follow-up (w)	Outcome	PI duration (year)
Cao et al. (17)	36/36	38.5 ± 14	0.56	AC	PLA	4w	SE, TST	1.2
Gao and Luo (18)	22/18	44.67 ± 11.26	0.55	AC	BZD	4w	PSQI	5.3
Li et al. (19)	30/30	18–70	NA	AC	BZD	4w	PSQI	NA
Zhang et al. (20)	33/33	51.7 ± 2.3	0.59	CBT	NBZD	6 M	PSQI	NA
Zhao et al. (21)	45/45	43.97 ± 9.32	0.6	AC	BZD	4w	PSQI	1
Xuan et al. (22)	24/22	48.96 ± 11.96	0.54	AC	BZD	4w	PSQI, TST	5.31
Li et al. (23)	20/20	50.0 ± 3.49	0.55	AC	PLA	4w	PSQI	0.72
	20/20	50.0 ± 3.00	0.58	BZD	PLA	4w	PSQI	0.72
	20/20	50.0 ± 3.49	0.58	AC	BZD	4w	PSQI	0.72
Jiang (24)	60/60	18–75	NA	RT	NBZD	4w	PSQI	NA
Feng et al. (25)	43/37	53.57 ± 16.56	0.68	rTMS	PLA	1w	PSQI	0.69
Tu et al. (26)	19/14	44.56 ± 8.05	0.55	AC	PLA	4w	PSQI	NA
Jespersen et al. (27)	19/19	51.13 ± 9.6	0.79	RT	PLA	4w	PSQI	NA
Jacobs et al. (28)	15/15	46.85 ± 8.9	0.70	RT	PLA	8w	SL, TST, SE	9.55
	15/15	46.00 ± 9.56	0.73	NBZD	PLA	8w	SL, TST, SE	9.35
	15/15	46.25 ± 8.61	0.70	RT	NBZD	8w	SL, TST, SE	10
Edinger et al. (29)	25/25	55.75 ± 10.77	0.80	CBT	PLA	6 W	TST, SE	13.9
	25/25	55.1 ± 9.77	0.80	RT	PLA	7 W	TST, SE	14
	25/25	55.15 ± 11.1	0.73	CBT	RT	8 W	TST, SE	13.1
Siu et al. (30)	105/110	67.3 ± 6.8	0.8	RT	PLA	12 W	PSQI	10.37
Yin et al. (31)	36/36	38.5 ± 14.0	0.56	AC	PLA	4 W	TST	NA
Lee et al. (32)	46/45	51.94 ± 8.8	0.75	AC	PLA	12 W	PSQI, TST, SE, SL	6.31
Ritterband et al. (33)	22/22	44.86 ± 11.03	0.77	CBT	PLA	9 W	TST, SE, SL	10.59
Yeung et al. (34)	30/30	48.0 ± 9.0	0.77	AC	PLA	3 W	PSQI, TST, SE, SL	9.3
Vanstraten et al. (35)	59/59	49.4 ± 12.9	0.70	CBT	PLA	6 W	SE, SL	11.8
Scharf et al. (36)	221/214	45.42 ± 11.28	0.61	BZD	PLA	3 M	TST, SL	NA
Feng et al. (37)	17/17	38.15 ± 10.83	0.65	CUP	PLA	4 W	PSQI	3.97
	16/17	41.15 ± 10.8	0.58	BZD	PLA	4 W	PSQI	3.97
	17/16	39.49 ± 10.92	0.58	CUP	BZD	4 W	PSQI	3.97
Yeung et al. (38)	70/70	42.1 ± 13.0	0.80	AC	PLA	5 W	TST, SE, SL	5.3
Khalsa and Goldstein (39)	20/20	42.15 ± 10.55	0.63	RT	SH	8 W	PSQI, TST, SE, SL	NA
Riemann et al. (40)	18/18	47.02 ± 10.73	0.42	BZD	PLA	4 W	PSQI, TST, SE, SL	NA
Passos et al. (41)	12/12	44.4 ± 8	0.79	RT	PLA	24H	TST, SE, SL	9.1
Lo et al. (42)	14/13	56.9 ± 4.66	1.00	AC	PLA	3 W	PSQI, TST, SE, SL	16.8
Morin et al. (43)	18/18	64.65 ± 7.20	0.69	CBT	PLA	8 W	TST, SE	16.6
	17/18	64.51 ± 6.68	0.71	NBZD	PLA	8 W	TST, SE	17
	18/17	64.25 ± 6.89	0.63	CBT	NBZD	8 W	TST, SE	15.8
Wu et al. (44)	19/17	38.8 ± 12.1	0.53	CBT	PLA	8 W	TST, SE, SL	0.5
	17/17	38.8 ± 12.1	0.53	NBZD	PLA	8 W	TST, SE, SL	0.5
	19/17	38.8 ± 12.1	0.53	CBT	NBZD	8 W	TST, SE, SL	0.5
Fu et al. (45)	37/37	52.5 ± 5.56	1.00	AC	PLA	3 W	TST, SE, SL	0.25
Espie et al. (46)	107/94	54.26 ± 14.9	0.68	CBT	PLA	2 W	TST, SE, SL	0.5
Abedian et al. (47)	37/36	51 ± 4.44	1.00	AP	PLA	4 W	PSQI, SE, SL	1
Huang et al. (48)	18/18	45.08 ± 11.09	0.50	PT	PLA	10d	PSQI	0.25
Tang et al. (49)	38/38	45 ± 9.98	0.66	AP	BZD	1 M	PSQI	1.67
Wang and Zhou (50)	35/35	53.98 ± 4.81	0.57	AP	AC	4 W	PSQI	4.31
Lin et al. (51)	46/44	67.05 ± 6.28	0.50	AC	CBT	8 W	PSQI	4.8
Wang et al. (52)	33/30	73 ± 6	0.59	AC	BZD	4 W	PSQI	1.2
Zhang et al. (53)	30/30	78.07 ± 2.98	0.42	RT	Wait List	8 W	PSQI	5.43
Xu et al. (54)	27/27	68.15 ± 7.25	0.44	CBT	BZD	4 W	PSQI	6.51
Liang (55)	35/35	68 ± 6	0.57	AC	BZD	1 M	PSQI	5.41
Yu and Gao (56)	28/28	71.8 ± 5.25	0.52	AC	BZD	4 W	PSQI	4.71
Christina et al. (57)	11/9	77.2 ± 8	0.65	CBT	SH	1 W	TST, SE, SL	10.6
Edinger et al. (58)	20/20	54.2 ± 13.7	0.125	CBT	SH	2 W	TST, SE, SL	10
Espie et al. (59)	55/55	49 ± 13.5	0.73	CBT	PLA	6 W	TST, SE, SL	5.62
Jernelov et al. (60)	44/43	47.9 ± 13.9	0.82	CBT	Wait List	1 W	TST, SE, SL	11.8
Kaldo et al. (61)	73/75	48.01 ± 15.38	0.78	CBT	PLA	8 W	TST, SE, SL	10.5
Lichstein et al. (62)	27/23	68.08 ± 6.98	0.72	RT	PLA	2 W	TST, SE, SL	9.17
Lovato et al. (63)	86/32	63.76 ± 6.45	0.5	CBT	Wait List	4 W	TST, SE, SL	5
Sivertsen et al. (64)	9/8	60.51 ± 5.54	0.38	CBT	NBZD	2 W	TST, SE, SL	14.81
	9/6	60.6 ± 4.53	0.53	CBT	PLA	2 W	TST, SE, SL	14.28
	8/6	61.51 ± 5.94	0.54	NBZD	PLA	2 W	TST, SE, SL	12.97
Wei et al. (65)	40/40	47.53 ± 12.71	0.65	AP	BZD	3 W	PSQI	2.1
Wei et al. (66)	40/40	40.15 ± 5.19	0.69	AP	BZD	10D	PSQI	0.17
Yang et al. (67)	31/32	46.51 ± 12.43	0.63	AP	BZD	2 W	PSQI	5.8
Zhuang (68)	50/50	48.49 ± 2.26	0.45	AP	BZD	3 W	PSQI	4.03
Wei et al. (69)	30/30	47.23 ± 12.58	0.53	AP	BZD	12D	PSQI	2.2

T, treatment group; C, control group; w, week; PSQI, Pittsburgh Sleep Quality Index; TST, Total Sleep Time; SE, Sleep Efficiency; SL, Sleep Latency; AC, Acupuncture; CBT, Cognitive Behavioral Therapy; AP, Acupressure; RT, Relaxation Therapy; CUP, Cupping Therapy; SH, Sleep Hygiene; rTMS, Repetitive Transcranial Magnetic Stimulation; BZD, Benzodiazepines; NBZD, Non-benzodiazepine Drugs; PLA, Placebo (sham acupuncture or sham treatment); Wait List, waiting list. ICD, Diagnosis Systems: International Classification of Diseases; DSM, Diagnostic and Statistical Manual of Mental Disorders; NA, Not Available.

### 3.3 Risk of Bias assessment using ROB 2.0

**Figure 2:**
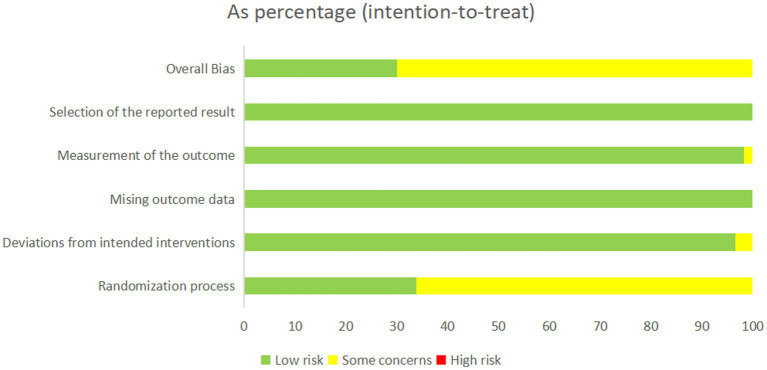
Risk of bias assessment using ROB 2.0 tool.

Study quality was evaluated using the Cochrane ROB 2.0 tool. In the randomization domain, 18 studies (33.9%) demonstrated low risk of bias, whereas 35 studies (66.1%) lacked clearly allocation concealment descriptions. All trials showed high adherence to protocol (low risk of deviation bias). Complete outcome data were available for all 53 studies, with appropriate measurement methods for primary outcomes using both subject and objective measures. No studies exhibited high risk of measurement bias. Selective reporting bias was minimal (16 studies, 30.1% low risk; 30 studies, 69.9% potential risk), as detailed in Figure 2 and Supplementary Table S9.

### 3.4 Evidence quality assessment

**Figure 3:**
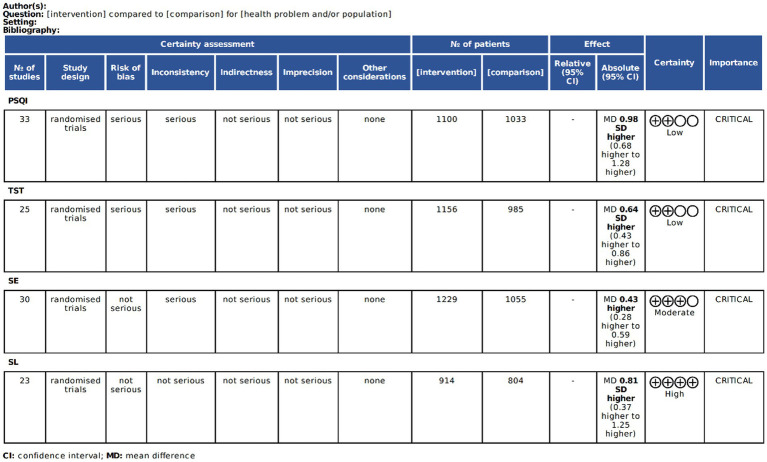
Results of evidence assessment using GRADE quality.

The GRADE system was applied to evaluate evidence quality for all primary outcomes. Final rating reflect comprehensive quality assessments across included studies (17–69) (Figure 3).

### 3.5 Pairwise meta-analysis results

**Figure 4:**
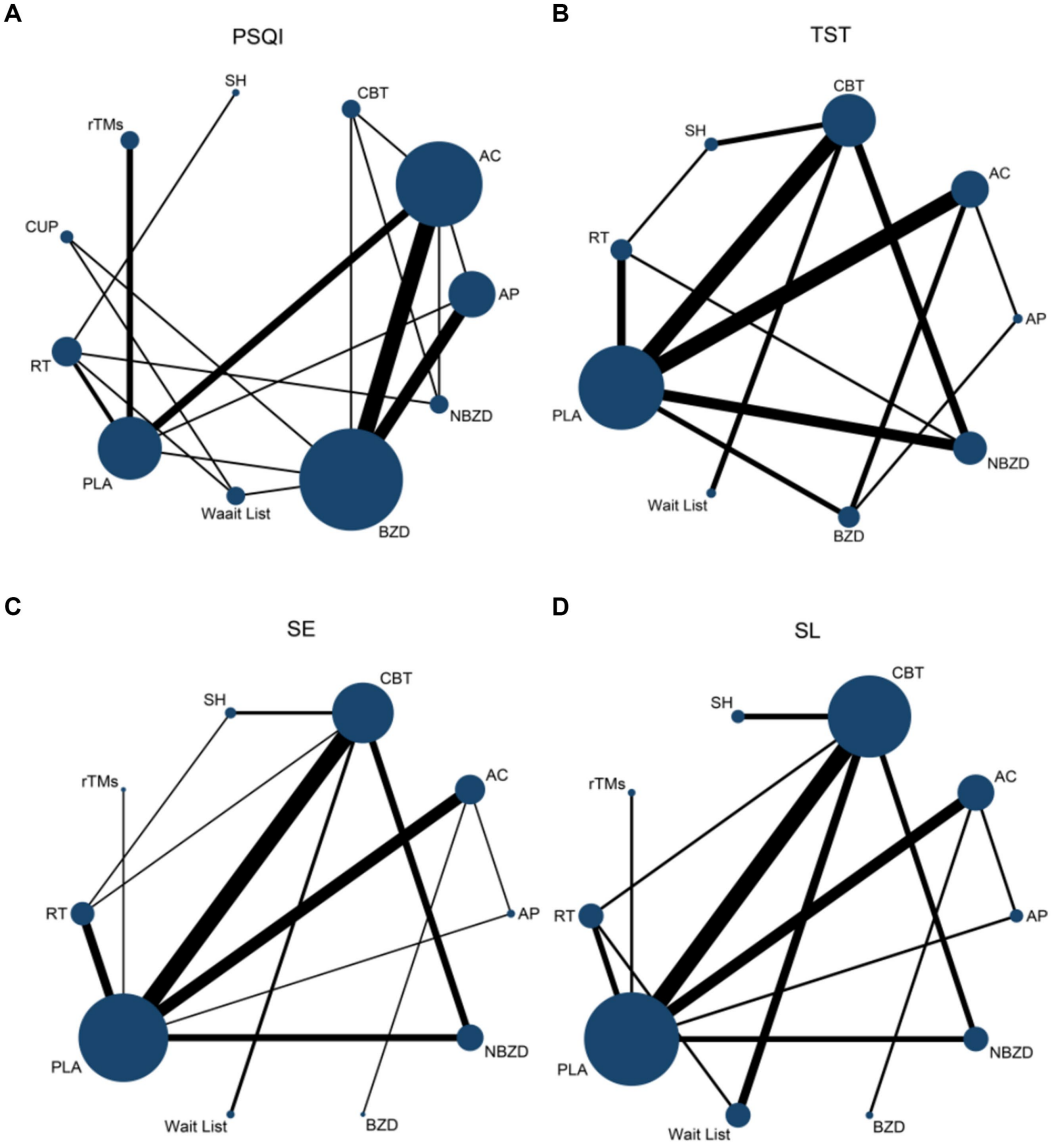
Network evidence map of pairwise comparison on PSQI **(A)**, TST **(B)**, SE **(C)**, and SL **(D)**. PSQI, Pittsburgh Sleep Quality Index; TST, Total Sleep Time; SE, sleep efficiency; SL, sleep latency; AC, Acupuncture; CBT, Cognitive Behavioral Therapy; AP, Acupressure/Massage; RT, Relaxation Therapy; CUP, Cupping Therapy: SH, Sleep Hygiene; rTMS, Repetitive Transcranial Magnetic Stimulation; BZD, Benzodiazepines; NBZD, Non-Benzodiazepines; PLA, Placebo (sham acupuncture or sham treatment); Wait List, Waiting List.

The pairwise meta-analysis results for PSQI, TST, SE, and SL are presented in the Supplementary Figures S1–S4, and Figure 4. Compared to PLA and the Wait List, both BZD (MD = −1.25, 95% CI: −2.48 to −0.02; MD = −1.90, 95% CI: −3.97 to 0.16) and NBZD (MD = −1.06, 95% CI: −2.95 to 0.83; MD = −1.71, 95% CI: −4.20 to 0.78) significantly reduced PSQI scores. Additionally, significant SE improvements were also observed for acupressure (MD = 1.27, 95% CI: 0.37 to 2.18; MD = 1.74, 95% CI: 0.69 to 2.80), acupuncture (MD = 0.88, 95% CI: 0.15 to 0.60; MD = 1.35, 95% CI: 0.51 to 2.20), CBT (MD = 1.14, 95% CI: 0.55 to 1.73; MD = 1.61, 95% CI: 0.67 to 2.55), and BZD (MD = 0.85, 95% CI: 0.10 to 1.59; MD = 1.32, 95% CI: 0.31 to 2.33), with CBT additionally demonstrating significant SL reduction (MD = −0.58, 95% CI: −0.85 to −0.31; MD = −0.63, 95% CI: −0.98 to −0.68).

Compared to SH, acupressure (MD = 1.48, 95% CI: 0.56 to 2.39; MD = −1.31, 95% CI: −2.20 to −0.42), CBT (MD = 1.34, 95% CI: 0.70 to 1.98; MD = −1.34, 95% CI: −2.02 to −0.66), CUP (MD = 0.82, 95% CI: 0.15 to 1.50; MD = −1.19, 95% CI: −2.00 to −0.37), and BZD (MD = 1.05, 95% CI: 0.28 to 1.59; MD = −1.32, 95% CI: −2.18 to −0.45) were all effective in improving SE and reducing SL. When compared to RT, acupressure (MD = 0.86, 95% CI: 0.23 to 1.49; MD = −0.55, 95% CI: −1.07 to −0.03) improved SE and shortened SL, while acupuncture showed a significant improvement in SE (MD = 0.47, 95% CI: 0.15 to 0.79), and CBT effectively shortened SL (MD = −0.58, 95% CI: −0.85 to −0.31).

Compared to the Wait List, CUP showed a significant improvement in SE (MD = 1.09, 95% CI: 0.11 to 2.07). Additionally, acupuncture (MD = −9.58, 95% CI: −11.15 to −8.00), acupressure (MD = −10.12, 95% CI: −11.78 to −8.46), CUP (MD = −10.00, 95% CI: −11.67 to −8.33), rTMS (MD = −9.65, 95% CI: −11.39 to −7.90), BZD (MD = −10.13, 95% CI: −11.83 to −8.43), PLA (MD = −9.52, 95% CI: −11.19 to −7.85), and RT (MD = −9.57, 95% CI: −11.19 to −7.56) were all effective in reducing SL. Moreover, acupuncture significantly extended TST compared to PLA, and shortened SL compared to acupressure and CBT.

### 3.6 Network meta-analysis findings

The network meta-analysis evaluated four efficacy indicators combining objective and subjective sleep measures. Thirty-two studies (*n* = 2,114) assessed PSQI scores, 25 studies (*n* = 2,451) measured TST, while SE and SL were evaluated in 2,284 and 1,718 patients, respectively (Table 2, panel A; Table 3, panel B).

**Table 2:** Results of network meta-analysis (Panel A).

AP	0.63 (−2.41,3.66)	1.35 (−2.10,4.80)	1.63 (−2.12,5.38)	–	–	1.55 (−2.06,5.15)	3.17 (−0.30,6.64)	1.16 (−2.42,4.74)	1.83 (−1.41,5.08)	1.61 (−2.45,5.67)
−0.26 (−1.39,0.87)	AC	0.73 (−0.91,2.36)	1.00 (−1.20,3.21)	–	–	0.92 (−1.02,2.87)	**2.54** **(0.86,4.22)**	0.53 (−1.37,2.43)	**1.21** **(0.05,2.36)**	0.99 (−1.71,3.68)
−0.39 (−2.26,1.48)	−0.14 (−1.78,1.51)	CBT	0.28 (−1.55,2.11)	–	–	0.20 (−1.60,2.00)	1.82 (−0.20,3.84)	−0.19 (−1.70,1.32)	0.48 (−0.68,1.64)	0.26 (−1.88,2.40)
−1.79 (−5.02,1.44)	−1.53 (−4.66,1.59)	−1.40 (−4.80,2.00)	SH	–	–	−0.08 (−2.13,1.97)	1.54 (−0.96,4.04)	−0.47 (−2.67,1.73)	0.21 (−1.67,2.08)	−0.02 (−2.83,2.80)
−1.40 (−3.44,0.65)	−1.14 (−3.02,0.74)	−1.00 (−3.43,1.42)	0.39 (−3.01,3.80)	rTMs	–	–	–	–	–	–
−0.36 (−3.02,2.31)	−0.10 (−2.70,2.50)	0.03 (−2.93,3.00)	1.43 (−2.41,5.27)	1.04 (−2.05,4.13)	CUP	–	–	–	–	–
−1.38 (−3.23,0.48)	−1.12 (−2.79,0.55)	−0.98 (−3.12,1.15)	0.41 (−2.23,3.06)	0.02 (−2.13,2.17)	−1.02 (−3.80,1.77)	RT	1.62 (−0.66,3.89)	−0.39 (−2.34,1.56)	0.29 (−1.28,1.85)	0.06 (−2.73,2.86)
−0.81 (−1.76,0.13)	−0.56 (−1.37,0.26)	−0.42 (−2.09,1.25)	0.98 (−2.17,4.12)	0.58 (−1.36,2.53)	−0.46 (−2.96,2.05)	0.56 (−1.14,2.27)	BZD	−2.01 (−4.25,0.23)	−1.33 (−2.98,0.32)	−1.56 (−4.50,1.39)
−1.00 (−2.98,0.98)	−0.75 (−2.48,0.99)	−0.61 (−2.51,1.29)	0.79 (−2.46,4.04)	0.39 (−2.02,2.81)	−0.64 (−3.63,2.34)	0.37 (−1.52,2.27)	−0.19 (−2.01,1.63)	NBZD	0.68 (−0.83,2.18)	0.45 (−2.17,3.07)
**−2.06** **(−3.45,-0.68)**	**−1.81** **(−2.93,-0.68)**	−1.67 (−3.57,0.22)	−0.27 (−3.32,2.78)	−0.67 (−2.17,0.84)	−1.71 (−4.41,0.99)	−0.69 (−2.22,0.84)	**−1.25** **(−2.48,-0.02)**	−1.06 (−2.95,0.83)	PLA	−0.22 ( −2.66,2.21)
−**2.71** **(−4.94,-0.49)**	−**2.46** **(−4.58,-0.34)**	−2.32 (−4.87,0.22)	−0.92 (−4.26,2.41)	−1.32 (−3.96,1.32)	−2.36 (−4.90,0.18)	−1.34 (−3.38,0.70)	−1.90 (−3.97,0.16)	−1.71 (−4.20,0.78)	−0.65 (−2.82,1.52)	Wait List

In panel A, the lower left corner shows the Pittsburgh Sleep Quality Index (PSQI), the upper right corner displays Total Sleep Time (TST); AC, Acupuncture; CBT, Cognitive Behavioral Therapy; AP, Acupressure/Massage; RT, Relaxation Therapy; CUP, Cupping Therapy; SH, Sleep Hygiene; rTMS, Repetitive Transcranial Magnetic Stimulation; BZD, Benzodiazepines; NBZD, Non-Benzodiazepines; PLA, Placebo (sham acupuncture or sham treatment); Wait List, Waiting List. Bold values in tables indicate statistically significant results (*p* < 0.05).

**Table 3:** Results of network meta-analysis (Panel B).

AP	−0.52 (−1.09,0.06)	0.06 (−0.58,0.70)	**−1.01** **(−1.86,-0.17)**	−0.51 (−1.44,0.42)	−0.40 (−1.15,0.35)	**−10.09** **(−11.80,-8.39)**	0.01 (−0.80,0.82)	**−0.58** **(−1.15,-0.01)**	−0.62 (−1.36,0.12)
−0.34 (−1.37,0.68)	AC	**0.58** **(0.06,1.10)**	−0.50 (−1.26,0.26)	0.01 (−0.84,0.86)	0.12 (−0.53,0.77)	**−9.58** **(−11.18,-7.98)**	0.52 (−0.19,1.24)	−0.07 (−0.50,0.37)	−0.10 (−0.74,0.54)
−0.70 (−1.72,0.31)	−0.36 (−0.83,0.11)	CBT	**−1.08** **(−1.67,-0.49)**	−0.57 (−1.36,0.22)	−0.46 (−0.97,0.05)	**−10.16** **(−11.84,-8.47)**	−0.05 (−0.63,0.52)	**−0.64** **(−0.94,-0.35)**	**−0.68** **(−1.06,-0.29)**
0.64 (−0.57,1.85)	**0.99** **(0.18,1.80)**	**1.35** **(0.65,2.05)**	SH	0.51 (−0.46,1.47)	0.62 (−0.02,1.25)	**−9.08** **(−10.85,-7.31)**	**1.02** **(0.21,1.83)**	0.43 (−0.19,1.06)	0.40 (−0.29,1.08)
−0.15 (−1.51,1.22)	0.20 (−0.83,1.22)	0.55 (−0.46,1.57)	−0.79 (−2.00,0.41)	rTMs	0.11 (−0.77,0.99)	**−9.58** **(−11.40,-7.77)**	0.52 (−0.41,1.45)	−0.07 (−0.80,0.66)	−0.11 (−0.97,0.76)
−0.18 (−1.24,0.88)	0.16 (−0.39,0.72)	**0.52** **(0.03,1.01)**	**−0.82** **(−1.57,-0.08)**	−0.03 (−1.09,1.02)	RT	−**9.70** (**−11.43,-7.97**)	0.41 (−0.33,1.14)	−0.18 (−0.67,0.30)	−0.22 (−0.80,0.36)
1.01 (−0.40,2.42)	**1.35** **(0.39,2.32)**	**1.71** **(0.64,2.79)**	0.36 (−0.90,1.62)	1.16 (−0.25,2.57)	**1.19** **(0.07,2.30)**	BZD	**10.10** **(8.35,11.86)**	**9.51** **(7.85,11.17)**	**9.48** **(7.75,11.20)**
−0.41 (−1.50,0.68)	−0.06 (−0.68,0.55)	0.29 (−0.21,0.79)	**−1.05** **(−1.90,-0.21)**	−0.26 (−1.35,0.83)	−0.23 (−0.87,0.42)	**−1.42** (**−2.56,-0.27**)	NBZD	**−0.59** **(−1.17,-0.01)**	−0.63 (−1.31,0.06)
0.02 (−0.94,0.99)	**0.37** **(0.02,0.72)**	**0.73** **(0.42,1.03)**	−0.62 (−1.35,0.11)	0.17 (−0.79,1.13)	0.20 (−0.23,0.63)	−0.99 (−2.02,0.04)	0.43 (−0.07,0.93)	PLA	−0.04 (−0.50,0.43)
0.44 (−0.79,1.66)	0.78 (−0.04,1.61)	**1.14** **(0.46,1.82)**	−0.21 (−1.18,0.77)	0.58 (−0.64,1.80)	0.62 (−0.22,1.45)	−0.57 (−1.84,0.70)	**0.84** **(0.00,1.69)**	0.41 (−0.33,1.16)	Wait List

In panel B, the lower left corner indicates Sleep Efficiency (SE), while the upper right corner represents Sleep Latency (SL); AC, Acupuncture; CBT, Cognitive Behavioral Therapy; AP, Acupressure/Massage; RT, Relaxation Therapy; CUP, Cupping Therapy; SH, Sleep Hygiene; rTMS, Repetitive Transcranial Magnetic Stimulation; BZD, Benzodiazepines; NBZD, Non-Benzodiazepines; PLA, Placebo (sham acupuncture or sham treatment); Wait List, Waiting List. Bold values in tables indicate statistically significant results (*p* < 0.05).

Compared to PLA and the Wait List, both acupressure (MD = -2.06, 95% CI -3.45 to −0.68; MD = -2.71, 95% CI -4.94 to −0.49) and acupuncture (MD = -1.81, 95% CI -2.93 to −0.68; MD = -2.46, 95% CI -4.58 to −0.34) significantly decreased PSQI scores. BZD also showed PSQI reduction versus PLA (MD = -1.25, 95% CI -2.48 to −0.02). For TST, acupuncture outperformed BZD (MD = 2.07, 95% CI 0.46–3.68).

For SE improvement, network meta-analysis revealed significant benefits across multiple interventions. Acupressure showed superior efficacy versus PLA (MD = 0.86, 95% CI: 0.23 to 1.49), Wait List (MD = 1.27, 95% CI: 0.37 to 2.18), SH (MD = 1.48, 95% CI: 0.56 to 2.39), and BZD (MD = 1.47, 95% CI: 0.69 to 2.80). CBT demonstrated comparable effects versus PLA (MD = 0.72, 95% CI: 0.45 to 1.00), Wait List (MD = 1.14, 95% CI: 0.55 to 1.73), SH (MD = 1.34, 95% CI: 0.70 to 1.98), and BZD (MD = 1.67, 95% CI: 0.61 to 2.55), as did acupuncture versus PLA (MD = 0.47, 95% CI: 0.15 to 0.79), Wait List (MD = 0.88, 95% CI: 0.15 to 1.60), SH (MD = 1.08, 95% CI: 0.35 to 1.82), and BZD (MD = 1.35, 95% CI: 0.51 to 2.20).

NBZD also significantly improved SE versus Wait List (MD = 0.85, 95% CI: 0.10 to 1.59), SH (MD = 1.05, 95% CI: 0.28 to 1.82), and BZD (MD = 1.32, 95% CI: 0.31 to 2.33). Furthermore, CBT outperformed RT (MD = 0.52, 95% CI: 0.08 to 0.95), while RT itself showed advantages over SH (MD = 0.82, 95% CI: 0.15 to 1.50) and BZD (MD = 1.09, 95% CI: 0.11 to 2.07).

For SL reduction, multiple interventions demonstrated significant efficacy versus comparators. CBT (MD = −1.34, 95% CI: −2.02 to −0.66; MD = −10.15, 95% CI: −11.79 to −8.52), acupressure (MD = −1.31, 95% CI: −2.20 to −0.42; MD = −10.12, 95% CI: −11.78 to −8.46), NBZD (MD = −1.32, 95% CI: −2.18 to −0.45; MD = −10.13, 95% CI: −11.83 to −8.43), RT (MD = −1.19, 95% CI: −2.00 to −0.37; MD = −10.00, 95% CI: −11.67 to −8.33), and PLA (MD = −0.76, 95% CI: −1.49 to −0.03; MD = −9.57, 95% CI: −11.19 to −7.96) all outperformed both SH and BZD.

Compared to acupuncture and PLA, CBT (MD = −0.58, 95% CI: −1.01 to −0.14; MD = −0.58, 95% CI: −0.85 to −0.31) and acupressure (MD = −0.54, 95% CI: −1.06 to −0.02; MD = −0.55, 95% CI: −1.07 to −0.03) showed greater SL reduction. Furthermore, rTMS (MD = −9.65, 95% CI: −11.39 to −7.90), acupuncture (MD = −9.58, 95% CI: −11.15 to −8.00), Wait List (MD = −9.25, 95% CI: −11.19 to −7.85), and SH (MD = −8.81, 95% CI: −10.58 to −7.05) were all superior to BZD for SL reduction. CBT also exceeded Wait List (MD = −0.63, 95% CI: −0.98 to −0.28), while NBZD surpassed PLA (MD = −0.56, 95% CI: −1.10 to −0.01) in shortening SL.

### 3.7 Inconsistency and heterogeneity analysis

Global inconsistency tests revealed significant heterogeneity across all outcomes (PSQI, TST, SE, and SL; all *p* < 0.05). Node-splitting analysis confirmed local inconsistencies (*p* < 0.05) in six triangular loops for PSQI (Supplementary Figure S5). The acupressure-PLA-BZD loop showed the highest inconsistency factor (IF = 5.327, 95% CI: 2.80, 7.86), followed by acupressure-acupuncture-PLA (IF = 3.594, 95% CI: 0.00, 10.62) and acupuncture-CBT-NBZD (IF = 3.392, 95% CI: 2.36, 4.42). Other loops demonstrated lower inconsistency: acupuncture-PLA-BZD (IF = 3.208, 95% CI: 0.00, 7.42), acupressure-acupuncture-BZD (IF = 1.476, 95% CI: 0.00, 3.92), and acupuncture-CBT-BZD (IF = 0.611, 95% CI: 0.00, 4.20). Inconsistency was confirmed in two loops (95% CI excluding 0), while four showed potential consistency (95% CI excluding 0), indicating variability between direct and indirect evidence.

For TST analysis, four triangular loops were formed, showing varying inconsistency levels. The acupuncture-PLA-BZD loop exhibited moderate inconsistency (IF = 4.878, 95% CI: 0.65, 9.11), followed by acupressure-acupuncture -BZD (IF = 3.912, 95% CI: 3.00, 4.82). Lower inconsistency appeared in CBT-PLA-NBZD (IF = 0.187, 95% CI: 0.00, 1.58) and RT-PLA-NBZD (IF = 0.154, 95% CI: 0.00, 2.46). Two loops demonstrated significant inconsistency (95% CI excluding 0), suggesting discordance between direct/indirect comparisons that warrants further investigation.

For SE analysis, four triangular loops were analyzed with distinct inconsistency patterns. The acupressure-acupuncture-RT loop showed the highest inconsistency (IF = 1.868, 95% CI: 0.59, 3.15). Three loops displayed minimal inconsistency: CBT-SH-CUP (IF = 0.451, 95% CI: 0.00, 1.09), CBT-RT-BZD (IF = 0.56, 95% CI: 0.00, 0.85), and CBT-CUP-RT (IF = 0.451, 95% CI: 0.00, 1.09). Two loops exhibited significant inconsistency (95% CI excluding 0), indicating potential divergence between evidence sources.

For SL analysis, four triangular loops were assessed. The acupressure-acupuncture-RT loop showed mild inconsistency (IF = 1.401, 95% CI: 0.21, 2.59), while three loops demonstrated minimal inconsistency: CBT-CUP-RT (IF = 0.654, 95% CI: 0.00, 1.48), CBT-RT-BZD (IF = 0.180, 95% CI: 0.00, 1.09), and CBT-CUP-PLA (IF = 0.062, 95% CI: 0.00, 1.16). Only one loop exhibited significant inconsistency (95% CI excluding 0), suggesting generally consistent direct and indirect comparisons.

Substantial heterogeneity was observed across all outcomes: PSQI (*I*^2^ = 91.8%), TST (*I*^2^ = 88.3%), SE (*I*^2^ = 69.3%), and SL (*I*^2^ = 96.6%). Visual inspection of funnel plots revealed symmetrical distributions for all outcomes, indicating low risk of publication bias (Figure 4).

### 3.8 SUCRA rankings of interventions

The average SUCRA values for each intervention are presented in the Supplementary Figures S6–S9. CBT demonstrated the highest efficacy for both reducing PSQI scores (84.4% probability) and improving SE (91.5%), while ranking first for shortening SL (85.8%). For TST extension, acupuncture showed the highest probability (84.8%), followed by NBZD (62.8%). NBZD also ranked second for SL reduction (82.5% probability). CBT maintained strong performance across all outcomes, ranking third for PSQI reduction (69.9%) and TST extension (54.6%), and second for SE improvement (89.2%). Acupuncture additionally ranked third for SE improvement (70.5% probability).

### 3.9 Sensitivity and subgroup analyses

The leave-one-out sensitivity analysis confirmed that while excluding individual studies slightly altered effect sizes and *p*-values (Supplementary Figure S10), the pooled effect direction and statistical significance remained consistent. This consistency indicates robust meta-analytic findings.

Subgroup analyses by treatment duration (Supplementary Figure S11) and disease course (Supplementary Figure S12) revealed substantial heterogeneity across studies (*I*^2^ ≥ 69%, *p* < 0.0001), suggesting significant variability in outcomes. This heterogeneity likely arises from differences in study design (e.g., inclusion criteria, intervention protocols, control groups) or patient characteristics (e.g., disease severity, comorbidities). Between-subgroup heterogeneity was statistically significant for TST and SL (*p* < 0.05), indicating that treatment duration influences these outcomes. Short-term treatment (0–5 weeks) worsened PSQI and SL scores, whereas long-term treatment (>10 weeks) showed more stable effects, particularly for SE, which exhibited low heterogeneity. Disease duration significantly affected PSQI, TST, and SL (*p* < 0.05). Shorter disease duration was associated with increased PSQI scores (worsened sleep quality), suggesting greater challenges in improving sleep during early disease stages. TST and SL varied across subgroups, with shorter disease duration linked to reduced TST or prolonged SL, highlighting prominent sleep initiation difficulties. In contrast, SE did not differ significantly between subgroups, implying that disease duration has a weaker impact on sleep efficiency than on other metrics, though the overall trend remained unfavorable.

### 3.10 Safety analysis

Among the 53 included studies, 23 (43.4%) reported adverse events. Medication-related adverse effects (6 studies) included hepatorenal dysfunction, cardiovascular events, xerostomia, and daytime somnolence. Acupuncture-related adverse events (2 studies) comprised transient local pain, bruising, and hand paresthesia, all resolving with continued treatment. One study reported sleep restriction in the CBT group. Four studies documented unspecified adverse events, while 10 studies reported no safety concerns. Overall, adverse event incidence was low, with acupuncture demonstrating particularly favorable safety due to its transient effects.

## 4 Discussion

PI remains a significant global public health challenge. This systematic review of 53 RCTs demonstrates that seven non-pharmacological interventions—compared against sham controls or pharmacotherapies—significantly improved core sleep parameters, including PSQI scores, TST, SE, and SL, supporting their role as first-line treatments for PI. These findings, while compelling, are tempered by the low-to-moderate certainty of evidence, primarily due to methodological limitations in the included studies.

Pairwise meta-analyses demonstrated that acupressure, acupuncture, and CBT were particularly effective in improving specific sleep parameters associated with PI, including PSQI scores, SE, SL, and TST. Among these, CBT showed the most pronounced effects in reducing PSQI scores and SL while enhancing SE, whereas acupuncture yield superior outcomes in extending TST. These results are consistent with previous findings (70), reinforcing CBT’s status as the benchmark non-pharmacological intervention for the treatment of PI. While PSQI, TST, SE, and SL are validated measures of sleep architecture, they do not assess key insomnia domains like cognitive hyperarousal or daytime impairment—a limitation inherent to our focus on sleep-specific outcomes. The PSQI’s inclusion of non-specific factors (e.g., sleep environment) may reduce its sensitivity for pure insomnia assessment compared to the ISI (71), which directly measures core symptoms. We recommend future trials adopt ISI to standardize insomnia-specific evaluation while maintaining PSQI for broader sleep quality assessment.

In contrast, BZDs showed limited efficacy, performing worse than waitlist controls in most sleep outcomes, with the exception of PSQI scores. This inferior performance likely reflects the well-documented risks of BZDs, such as tolerance, dependence, and withdrawal symptoms (72). Although NBZDs were effective in increasing TST and shortening SL, concerns remain regarding their adverse effect profile. Notably, non-pharmacological interventions—particularly CBT—were linked to significantly fewer side effects and demonstrated better safety and long-term tolerability, making them more appropriate for chronic PI, where pharmacological treatments may exacerbate cognitive impairment, mood disturbances, or rebound insomnia.

Subgroup and sensitivity analyses further validated the robustness of the findings. Specifically, patients with long-standing PI (duration >10 years) and those undergoing prolonged treatment exhibited greater therapeutic benefits alongside reduced heterogeneity, suggesting that non-pharmacological interventions may offer superior efficacy in chronic PI management. These results support a paradigm shift in clinical practice, advocating for the reclassification of non-pharmacological therapies—traditionally regarded as adjunctive—to first-line treatment status in evidence-based guidelines.

Nevertheless, this review focused solely on monotherapy approaches. Future studies should investigate synergistic effects of combined interventions (e.g., CBT plus acupuncture) to optimize clinical outcomes. Additionally, pragmatic trials focusing on implementation are needed to evaluate real-world barriers like cost, availability, and provider training—that may influence accessibility and adherence. These studies will be essential for developing practical, scalable care models for PI.

### 4.1 Strengths and limitations

The strengths of this study include the use of guideline-recommended therapeutic approaches as the basis for literature searches, offering a strong basis for clinical decision-making when selecting appropriate treatment options. Sensitivity analyses confirmed the robustness of the results, as no directional changes were observed, supporting the reliability of the findings. However, substantial heterogeneity remained despite performing subgroup analyses based on treatment duration and disease course.

Variability in study design, inclusion and exclusion criteria, baseline characteristics (e.g., average age and geographic location), and trial duration likely contributed to these inconsistencies. Furthermore, the use of different scoring systems across the included RCTs limited our ability to fully resolve the observed heterogeneity.

While network meta-analysis provides a powerful framework for comparing multiple interventions indirectly, its validity depends critically on the quality and methodological consistency of included studies. Several limitations should be acknowledged. First, the analysis included 53 eligible RCTs, limiting statistical power and generalizability. Many trials had small sample sizes and exhibited methodological heterogeneity, particularly in intervention protocols, blinding procedures, and allocation concealment. Although most studies employed adequate randomization, the inherent challenges of blinding in non-pharmacological research—coupled with a lack of validation regarding its success—may have introduced performance and detection biases, potentially compromising outcome objectivity. Further limitations arise from inconsistencies in outcome measurement and reporting. Critical metrics of sleep maintenance, such as WASO and early morning awakening, were excluded due to heterogeneous definitions and insufficient standardized data across studies. The PSQI, while a widely accepted tool, does not specifically quantify WASO, thereby limiting its sensitivity in detecting sleep fragmentation. These discrepancies underscore the pressing need for uniform outcome measures in insomnia research to facilitate cross-study comparisons and enhance clinical relevance. To address these gaps, future investigations should prioritize large-scale, rigorously RCTs featuring robust blinding procedures, transparent reporting standards, and harmonized outcome assessments. Such efforts would strengthen the evidence base and yield more definitive guidance for clinical practice.

## 5 Conclusion

Non-pharmacological interventions demonstrated superior efficacy compared with pharmacological approaches for improving multiple sleep parameters in patients with PI. These therapies significantly increased TST, enhanced SE, reduced SL, and lowered PSQI scores. Additionally, they exhibited excellent safety profiles and high patient acceptability. Among all modalities examined, CBT, acupuncture, and acupressure emerged as the most effective options and should be considered first-line treatments.

Despite the promising results, several limitations should be acknowledged that may affect the generalizability and interpretation of the findings. Most of the included trials used the PSQI as the primary outcome measure. Although widely used, the PSQI assesses a broad range of sleep-related domains and may lack specificity for core insomnia symptoms. Notably, the PSQI does not directly evaluate key features such as cognitive hyperarousal or daytime impairment. In addition, other clinically relevant indicators—such as WASO and early morning awakening—were excluded due to inconsistent reporting and methodological heterogeneity. These limitations may reduce both the interpretability and generalizability of the results.

To enhance measurement precision and comparability across studies, future trials should employ more insomnia-specific instruments, such as the ISI, which directly assesses the severity of insomnia symptoms and their impact on daytime functioning. Moreover, large-scale, rigorously designed RCTs using standardized outcome measures are needed to strengthen the evidence base and support future guideline development.

## Data Availability

The original contributions presented in the study are included in the article/Supplementary material, further inquiries can be directed to the corresponding author/s.

## Glossary

PI: Primary insomnia
RCTs: randomized controlled trials
CBT: cognitive behavioral therapy
PSQI: Pittsburgh Sleep Quality Index
TST: total sleep time
SE: sleep efficiency
SL: sleep latency
ROB: Cochrane Risk of Bias
RR: relative risk
CI: confidence interval
MD: Mean difference
SUCRA: Surface under the Cumulative Ranking
RT: Relaxation Therapy
CUP: Cupping Therapy
SH: Sleep Hygiene
rTMS: Repetitive Transcranial Magnetic Stimulation
BZD: Benzodiazepines
NBZD: Non-Benzodiazepines
PLA: Placebo (sham acupuncture or sham treatment)
Wait List: Waiting List
IF: inconsistency factor
