# Supplementary material for: Efficacy and safety of non-pharmacological therapies for primary insomnia: a network meta-analysis
Source: Front Neurol. 2025 Jul 29;16:1607903. doi: 10.3389/fneur.2025.1607903 (PMC12344310; doi:10.3389/fneur.2025.1607903)
Supplement: Supplementary file 1 [file Data_Sheet_1.pdf]

## Supplementary material | Table of Contents

**Supplementary Table S1**. Search strategy for PubMed database....................................................2

**Supplementary Table S2.** Search strategy for Cochrane Library database....................................4

**Supplementary Table S3.** Search strategy for EMBASE database..................................................6

**Supplementary Table S4.** Search strategy for Web of Science database.........................................8

**Supplementary Table S5.** Search strategy for CNKI database.......................................................9

**Supplementary Table S6.** Search strategy for Wanfang database..................................................10

**Supplementary Table S7.** Search strategy for VIP database...........................................................11

**Supplementary Table S8.** Search strategy for SinoMed database.................................................12

**Supplementary Table S9.** Risk of bias assessment using ROB 2.0 tool........................................13

**Supplementary Figure S1.** Results of pairwise comparison on PSQI...........................................16

**Supplementary Figure S2.** Results of pairwise comparison on TST.............................................17

**Supplementary Figure S3.** Results of pairwise comparison on SE...............................................18

**Supplementary Figure S4.** Results of pairwise comparison on SL...............................................19

**Supplementary Figure S5.** Funnel plots for PSQI, TST, SE, and SL............................................20

**Supplementary Figure S6.** Average SUCRA values for PSQI......................................................21

**Supplementary Figure S7.** Average SUCRA values for TST........................................................21

**Supplementary Figure S8.** Average SUCRA values for SE..........................................................22

**Supplementary Figure S9.** Average SUCRA values for SL...........................................................22

**Supplementary Figure S10.** Sensitivity analysis results.................................................................23

**Supplementary Figure S11.** Subgroup analysis based on treatment duration...............................24

**Supplementary Figure S12.** Subgroup analysis based on disease course.....................................25

**Supplementary Table S1.** Search strategy for PubMed database

| No. | Search terms | Results |
|---|---|---|
| #1 | "Sleep Initiation and Maintenance Disorders"[MeSH Terms] | 17772 |
| #2 | "primary insomnia"[Title/Abstract] OR "DIMS"[Title/Abstract] | 1362 |
| #3 | #1 OR #2 | 18336 |
| #4 | "Cognitive Behavioral Therapy"[MeSH Terms] OR "Behavior Therapy"[MeSH Terms] | 89349 |
| #5 | "relaxation"[Title/Abstract] OR "biofeedback"[Title/Abstract] OR "mindfulness"[Title/Abstract] | 154694 |
| #6 | #4 OR #5 | 228213 |
| #7 | "Benzodiazepines"[MeSH Terms] | 69671 |
| #8 | "benzodiazepine compounds"[Title/Abstract] OR "BZ"[Title/Abstract] OR "BZRAs"[Title/Abstract] OR "NBZRAs"[Title/Abstract] | 4596 |
| #9 | #7 OR #8 | 73538 |
| #10 | "antidepressant"[Title/Abstract] | 52990 |
| #11 | "Melatonin"[Title/Abstract] | 29867 |
| #12 | "antihistamine"[Title/Abstract] | 6179 |
| #13 | "orexin"[Title/Abstract] | 5329 |
| #14 | #10 OR #11 OR #12 OR #13 | 93650 |
| #15 | "Phototherapy"[MeSH Terms] | 53356 |
| #16 | "light therapy"[Title/Abstract] OR "photoradiation therapy"[Title/Abstract] | 2986 |
| #17 | #15 OR #16 | 54761 |
| #18 | "Exercise Therapy"[MeSH Terms] OR "Exercise"[MeSH Terms] | 283705 |
| #19 | "physical exercise"[Title/Abstract] | 20668 |
| #20 | #18 OR #19 | 293854 |
| #21 | "Acupuncture Therapy"[MeSH Terms] OR "Acupuncture"[MeSH Terms] | 29941 |
| #22 | "acupuncture ear"[Title/Abstract] OR "Electroacupuncture"[Title/Abstract] OR "fire needle"[Title/Abstract] OR "dry needle"[Title/Abstract] | 6962 |
| #23 | #21 OR #22 | 31388 |
| #24 | "Acupressure"[Title/Abstract] | 1556 |
| #25 | "Aromatherapy"[Title/Abstract] | 1663 |
| #26 | "foot reflexology"[Title/Abstract] | 137 |
| #27 | "Homeopathy"[Title/Abstract] | 3728 |
| #28 | "meditative movement therapy"[Title/Abstract] | 3 |
| #29 | "Moxibustion"[Title/Abstract] | 3674 |
| #30 | #24 OR #25 OR #26 OR #27 OR #28 OR #29 | 10554 |
| #31 | "Music Therapy"[MeSH Terms] | 4312 |

| | | |
|---|---|---|
| #32 | "Musicotherapy"[Title/Abstract] OR "melodiotherapy"[Title/Abstract] | 19 |
| #33 | #31 OR #32 | 4325 |
| #34 | "Yoga"[MeSH Terms] OR "Muscle Stretching Exercises"[MeSH Terms] | 5799 |
| #35 | "relaxed stretching"[Title/Abstract] | 1 |
| #36 | #34 OR #35 | 5799 |
| #37 | "random*"[Title/Abstract] | 1443118 |
| #38 | "randomized controlled trial"[Publication Type] | 599205 |
| #39 | #40 OR #41 | 1573279 |
| #40 | #6 OR #9 OR #14 OR #17 OR #20 OR #23 OR #30 OR #33 OR #36 OR #39 | 837904 |
| #41 | #3 AND #39 AND #40 | 1782 |

**Supplementary Table S2.** Search strategy for Cochrane Library database

| No. | Search terms | Results |
|---|---|---|
| #1 | MeSH descriptor: [Sleep Initiation and Maintenance Disorders] explode all trees | 3302 |
| #2 | (primary insomnia):ti,ab,kw OR (DIMS):ti,ab,kw | 5750 |
| #3 | #1 OR #2 | 8132 |
| #4 | MeSH descriptor: [Cognitive Behavioral Therapy] explode all trees | 12999 |
| #5 | MeSH descriptor: [Behavior Therapy] explode all trees | 23140 |
| #6 | (relaxation):ti,ab,kw OR (biofeedback):ti,ab,kw OR (mindfulness):ti,ab,kw | 26582 |
| #7 | #4 OR #5 OR #6 | 44087 |
| #8 | MeSH descriptor: [Benzodiazepines] explode all trees | 10606 |
| #9 | (benzodiazepine compounds):ti,ab,kw OR (BZ):ti,ab,kw OR (BZRAs):ti,ab,kw OR (NBZRAs):ti,ab,kw | 306 |
| #10 | #8 OR #9 | 10781 |
| #11 | antidepressant | 12276 |
| #12 | Melatonin | 3794 |
| #13 | antihistamine | 2046 |
| #14 | orexin | 455 |
| #15 | #11 OR #12 OR #13 OR #14 | 18374 |
| #16 | MeSH descriptor: [Phototherapy] explode all trees | 4432 |
| #17 | (light therapy):ti,ab,kw OR (photoradiation therapy):ti,ab,kw | 11316 |
| #18 | #17 OR #18 | 13122 |
| #19 | MeSH descriptor: [Exercise Therapy] explode all trees | 19663 |
| #20 | MeSH descriptor: [Exercise] explode all trees | 38402 |
| #21 | (physical exercise):ti,ab,kw | 50659 |
| #22 | #19 OR #20 OR #21 | 77156 |
| #23 | MeSH descriptor: [Acupuncture Therapy] explode all trees | 6450 |
| #24 | MeSH descriptor: [Acupuncture] explode all trees | 713 |
| #25 | MeSH descriptor: [Electroacupuncture] explode all trees | 1158 |
| #26 | MeSH descriptor: [Acupuncture, Ear] explode all trees | 241 |
| #27 | (dry needle):ti,ab,kw OR (fire needle):ti,ab,kw | 626 |
| #28 | #23 OR #24 OR #25 OR #26 OR #27 | 7560 |
| #29 | Acupressure | 2155 |
| #30 | Aromatherapy | 1513 |
| #31 | foot reflexology | 488 |
| #32 | Homeopathy | 1041 |
| #33 | meditative movement therapy | 29 |
| #34 | Moxibustion | 6443 |
| #35 | #29 OR #30 OR #31 OR #32 OR #33 OR #34 | 11419 |
| #36 | MeSH descriptor: [Music Therapy] explode all trees | 1233 |
| #37 | (Musicotherapy):ti,ab,kw OR (melodiotherapy):ti,ab,kw | 14 |

| | | |
|---|---|---|
| #38 | #36 OR #37 | 1247 |
| #39 | MeSH descriptor: [Yoga] explode all trees | 1210 |
| #40 | MeSH descriptor: [Muscle Stretching Exercises] explode all trees | 785 |
| #41 | (relaxed stretching):ti,ab,kw | 75 |
| #42 | #39 OR #40 OR #41 | 2048 |
| #43 | MeSH descriptor: [Randomized Controlled Trial] explode all trees | 25743 |
| #44 | (random*):ti,ab,kw | 1256032 |
| #45 | #43 OR #44 | 1256032 |
| #46 | #7 OR #10 OR #15 OR #18 OR #28 OR #35 OR #38 OR #42 OR #45 | 178247 |
| #47 | #3 AND #45 AND #46 | 2118 |

**Supplementary Table S3.** Search strategy for EMBASE database

| No. | Search terms | Results |
|---|---|---|
| #1 | 'primary insomnia'/exp | 799 |
| #2 | 'sleep initiation and maintenance disorders':ti,kw,ab OR 'dims':ti,ab,kw | 900 |
| #3 | #1 OR #2 | 1691 |
| #4 | 'cognitive behavioral therapy'/exp | 27343 |
| #5 | 'behavior therapy'/exp | 76513 |
| #6 | 'relaxation':ab,ti OR 'biofeedback':ab,ti OR 'mindfulness':ab,ti | 174018 |
| #7 | #4 OR #5 OR #6 | 245716 |
| #8 | 'benzodiazepine'/exp | 32071 |
| #9 | 'benzodiazepine compounds':ab,ti OR 'bz':ti,ab OR 'bzras':ti,ab | 5461 |
| #10 | #8 OR #9 | 37259 |
| #11 | 'antidepressant':ti,ab | 73489 |
| #12 | 'melatonin':ti,ab | 36580 |
| #13 | 'antihistamine':ti,ab | 9262 |
| #14 | 'orexin':ti,ab | 6976 |
| #15 | #11 OR #12 OR #13 OR #14 | 125309 |
| #16 | 'chinese medicine'/exp | 73693 |
| #17 | 'drugs, chinese herbal':ti,ab OR 'medicine, chinese traditional':ti,ab OR 'chinese drugs, plant':ti,ab OR 'chinese herbal medicine':ti,ab | 6292 |
| #18 | #16 OR #17 | 76493 |
| #19 | 'phototherapy'/exp | 115423 |
| #20 | 'light therapy':ti,ab OR 'photoradiation therapy':ti,ab | 3614 |
| #21 | #19 OR #20 | 116040 |
| #22 | 'exercise'/exp | 442682 |
| #23 | 'exercise therapy':ab,ti OR 'physical exercise':ab,ti | 33020 |
| #24 | #22 OR #23 | 452967 |
| #25 | 'acupuncture'/exp | 57395 |
| #26 | 'electroacupuncture':ab,ti OR 'dry needle':ab,ti OR 'fire needle':ab,ti OR 'acupoint':ab,ti | 12425 |
| #27 | #25 OR #26 | 58592 |
| #28 | 'acupressure':ab,ti | 2020 |
| #29 | 'aromatherapy':ab,ti | 2324 |
| #30 | 'foot reflexology':ab,ti | 215 |
| #31 | 'homeopathy':ab,ti | 4754 |
| #32 | 'meditative movement therapy':ab,ti | 2 |
| #33 | 'moxibustion':ab,ti | 4632 |
| #34 | #28 OR #29 OR #30 OR #31 OR #32 OR #33 | 13648 |
| #35 | 'music therapy'/exp | 9000 |
| #36 | 'musicotherapy':ti,ab OR ':melodiotherapy':ti,ab | 85 |
| #37 | #35 OR #36 | 9024 |

| | | |
|---|---|---|
| #38 | 'yoga'/exp OR 'muscle stretching exercises'/exp | 16083 |
| #39 | 'relaxed stretching':ti,ab | 0 |
| #40 | #38 OR #39 | 16083 |
| #41 | 'randomized controlled trial'/exp | 780683 |
| #42 | 'random*':ab,ti | 1957635 |
| #43 | #41 OR #42 | 2074075 |
| #44 | #7 OR #10 OR #15 OR #18 OR #21 OR #24 OR #27 OR #34 OR #37 OR #40 | 1094934 |
| #45 | #3 AND #43 AND #44 | 171 |

**Supplementary Table S4.** Search strategy for Web of Science database

| No. | Search terms | Results |
|---|---|---|
| #1 | TS=((primary insomnia) OR (sleep initiation and maintenance disorders) OR (DIMS)) | 30667 |
| #2 | TS=((Behavior Therapy) OR (Cognitive Behavioral Therapy) OR (relaxation) OR (biofeedback) OR (mindfulness)) | 626350 |
| #3 | TS=((benzodiazepine) OR (benzodiazepine compounds) OR (BZ) OR (BZRAs) OR (NBZRAs)) | 39115 |
| #4 | TS=(antidepressant ) | 89276 |
| #5 | TS=(melatonin ) | 43307 |
| #6 | TS=(antihistamine ) | 11860 |
| #7 | TS=(orexin ) | 7737 |
| #8 | TS=((phototherapy ) OR (light therapy) OR (photoradiation therapy) ) | 82500 |
| #9 | TS=((Exercise) OR (Exercise Therapy) OR (physical exercise)) | 570837 |
| #10 | TS=((Acupuncture) OR (Acupuncture Therapy) OR (Acupuncture, Ear) OR (Electroacupuncture) OR (dry needle) OR (fire needle) OR (acupoint) ) | 34841 |
| #11 | TS=(acupressure ) | 1873 |
| #12 | TS=(aromatherapy ) | 2222 |
| #13 | TS=(foot reflexology ) | 270 |
| #14 | TS=(homeopathy ) | 3729 |
| #15 | TS=(meditative movement therapy ) | 45 |
| #16 | TS=(moxibustion ) | 2295 |
| #17 | TS=((music therapy ) OR (musicotherapy) OR (melodiotherapy)) | 8389 |
| #18 | TS=((yoga ) OR (muscle stretching exercises) OR (relaxed stretching)) | 15024 |
| #19 | TS=((random*) OR (Randomized Controlled Trial)) | 2335009 |
| #20 | #2 OR #3 OR #4 OR #5 OR #6 OR #7 OR #8 OR #9 OR #10 OR #11 OR #12 OR #13 OR #14 OR #15 OR #16 OR #17 OR #18 | 1543165 |
| #21 | #1 AND #19 AND #20 | 1484 |

**Supplementary Table S5.** Search strategy for CNKI database

| No. | Search terms | Results |
|---|---|---|
| #1 | TKA=‘原发性失眠’ OR TKA=‘特发性失眠’ OR TKA=‘心理生理性失眠’ OR TKA=‘老年性失眠’ OR TKA=‘睡眠障碍’ | 75459 |
| #2 | TKA = '行为治疗' OR TKA = '行为疗法 ' OR TKA = '认知疗法' OR TKA = '放松疗法' OR TKA = '放松训练' OR TKA = '生物反馈' OR TKA = '正念减压' | 65097 |
| #3 | TKA =‘苯二氮卓’ OR TKA=‘BZ’ OR TKA=‘BZRAs’ OR TKA=‘抗抑郁’ OR TKA=‘抗组胺’ OR TKA=‘褪黑素’ OR TKA=‘食欲素’ | 41996 |
| #4 | TKA = '运动疗法' OR TKA = '运动' | 2905797 |
| #5 | TKA = ‘光照疗法’ OR TKA=‘光照’ | 145233 |
| #6 | TKA = '针灸' OR TKA = '针刺' OR TKA = '针法' OR TKA = '火针' OR TKA = '耳针' OR TKA = '电针' OR TKA = '干针' OR TKA = ‘穴位按摩’ OR TKA=‘推拿’ | 284289 |
| #7 | KA =‘芳香疗法’ OR TKA=‘精油’ OR TKA=‘香薰’ OR TKA=‘薰洗’ | 11204 |
| #8 | TKA =‘足部反射疗法’ OR TKA=‘足部穴位’ OR TKA=‘足部按摩 ’ | 766 |
| #9 | TKA =‘顺势疗法’ OR TKA=‘冥想运动疗法’ OR TKA=‘功法’ OR TKA=‘太极’ OR TKA=‘八段锦’ | 13000 |
| #10 | TKA = '艾灸' OR TKA = '隔物灸' OR TKA = '艾条灸' OR TKA = '雀啄灸' OR TKA = '回旋灸' OR TKA = '麦粒灸' OR TKA = '瘢痕灸' OR #12 TKA = '悬起灸' OR TKA = '艾炷灸' OR TKA = '热敏灸' | 19052 |
| #11 | TKA = ‘音乐疗法’ OR TKA=‘音乐’ | 342381 |
| #12 | TKA = ‘瑜伽’ OR TKA=‘舒展运动’ OR TKA=‘放松’ | 50437 |
| #13 | TKA = '随机对照' OR TKA = '随机' OR TKA = 'RCT' | 3649722 |
| #14 | #2 OR #3 OR #4 OR #5 OR #6 OR #7 OR #8 OR #9 OR #10 OR #11 OR #12 | 4876877 |
| #15 | #1 AND #13 AND #14 | 1554 |

**Supplementary Table S6.** Search strategy for Wanfang database

| No. | Search terms | Results |
|---|---|---|
| #1 | 题名或关键词:(原发性失眠 OR 特发性失眠 OR 心理生理性失眠 OR 老年性失眠 OR 睡眠障碍 ) | 13014 |
| #2 | 主题:(行为治疗 OR 行为疗法 OR 认知疗法 OR 放松疗法 OR 放松训练 OR 生物反馈 OR 正念减压 ) | 26645 |
| #3 | 主题:(苯二氮卓 OR BZ OR BZRAs OR 抗抑郁 OR 抗组胺 OR 褪黑素 OR 食欲素 ) | 47463 |
| #4 | 主题:(运动疗法 OR 运动 ) | 790 |
| #5 | 主题:(光照疗法 OR 光照 ) | 92049 |
| #6 | 主题:(针灸 OR 针刺 OR 针法 OR 火针 OR 耳针 OR 电针 OR 干针 OR 穴位) | 622441 |
| #7 | 主题:(芳香疗法 OR 精油 OR 香薰 OR 薰洗) | 35577 |
| #8 | 主题:(足部反射疗法 OR 足部穴位 OR 足部按摩) | 2021 |
| #9 | 主题:(顺势疗法 OR 冥想运动疗法 OR 功法 OR 太极 OR 八段锦) | 2910528 |
| #10 | 主题:(艾灸 OR 隔物灸 OR 艾条灸 OR 雀啄灸 OR 回旋灸 OR 麦粒灸 OR 瘢痕灸 OR 悬起灸 OR 艾炷灸 OR 热敏灸) | 18836 |
| #11 | 主题:(音乐疗法 OR 音乐) | 674747 |
| #12 | 主题:(瑜伽 OR 舒展运动 OR 放松) | 73356 |
| #13 | 主题:(随机对照 OR 随机 OR RCT) | 2720724 |
| #14 | #2 OR #3 OR #4 OR #5 OR #6 OR #7 OR #8 OR #9 OR #10 OR #11 OR #12 | 4792067 |
| #15 | #1 AND #13 AND #14 | 1648 |

**Supplementary Table S7.** Search strategy for VIP database

| No. | Search terms | Results |
|---|---|---|
| #1 | M=(原发性失眠 OR 特发性失眠 OR 心理生理性失眠 OR 老年性失眠 OR 睡眠障碍 ) | 13,491 |
| #2 | M=(行为治疗 OR 行为疗法 OR 认知疗法 OR 放松疗法 OR 放松训练 OR 生物反馈 OR 正念减压 ) | 17,652 |
| #3 | M=(苯二氮卓 OR BZ OR BZRAs OR 抗抑郁 OR 抗组胺 OR 褪黑素 OR 食欲素 ) | 15,321 |
| #4 | M=(运动疗法 OR 运动 ) | 6,714 |
| #5 | M=(光照疗法 OR 光照 ) | 21,638 |
| #6 | M=(针灸 OR 针刺 OR 针法 OR 火针 OR 耳针 OR 电针 OR 干针 OR 穴位) | 209,101 |
| #7 | M=(芳香疗法 OR 精油 OR 香薰 OR 薰洗) | 8,926 |
| #8 | M=(足部反射疗法 OR 足部穴位 OR 足部按摩) | 902 |
| #9 | M=(顺势疗法 OR 冥想运动疗法 OR 功法 OR 太极 OR 八段锦) | 27,880 |
| #10 | M=(艾灸 OR 隔物灸 OR 艾条灸 OR 雀啄灸 OR 回旋灸 OR 麦粒灸 OR 瘢痕灸 OR 悬起灸 OR 艾炷灸 OR 热敏灸) | 13,140 |
| #11 | M=(音乐疗法 OR 音乐) | 402,976 |
| #12 | M=(瑜伽 OR 舒展运动 OR 放松) | 19,709 |
| #13 | U=(随机对照 OR 随机 OR RCT) | 2,272,274 |
| #14 | #2 OR #3 OR #4 OR #5 OR #6 OR #7 OR #8 OR #9 OR #10 OR #11 OR #12 OR #13 | 1,009,937 |
| #15 | #1 AND #13 AND #14 | 815 |

**Supplementary Table S8. Search strategy for SinoMed database**

| No. | Search terms | Results |
|---|---|---|
| #1 | "入睡和睡眠障碍"[加权:扩展] OR "原发性失眠"[常用字段:智能] OR "特发性失眠"[常用字段:智能] OR "心理生理性失眠"[常用字段:智能] OR "老年性失眠"[常用字段:智能] | 37120 |
| #2 | "行为疗法"[加权:扩展] OR "认知疗法"[加权:扩展] OR "行为治疗"[常用字段:智能] OR "放松疗法"[常用字段:智能] OR "放松训练"[常用字段:智能] OR "生物反馈"[常用字段:智能] OR "正念减压"[常用字段:智能] | 20294 |
| #3 | "苯二氮卓"[常用字段:智能] OR "BZ"[常用字段:智能] OR "BZRAs"[常用字段:智能] OR "抗抑郁"[常用字段:智能] OR "抗组胺"[常用字段:智能] OR "褪黑素"[常用字段:智能] OR "食欲素"[常用字段:智能] | 22503 |
| #4 | "运动疗法"[加权:扩展] OR "运动"[常用字段:智能] | 318397 |
| #5 | "光照疗法"[加权:扩展] OR "光照"[常用字段:智能] | 18398 |
| #6 | "针灸疗法"[加权:扩展] OR "针灸"[常用字段:智能] OR "针刺"[常用字段:智能] OR "针法"[常用字段:智能] OR "火针"[常用字段:智能] OR "耳针"[常用字段:智能] OR "电针"[常用字段:智能] OR "干针"[常用字段:智能] OR "穴位"[常用字段:智能] | 299663 |
| #7 | "芳香疗法"[加权:扩展] OR "精油"[常用字段:智能] OR "香薰"[常用字段:智能] OR "薰洗"[常用字段:智能] | 14065 |
| #8 | "足部反射疗法"[加权:扩展] OR "足部穴位"[常用字段:智能] OR "足部按摩"[常用字段:智能] | 283 |
| #9 | "顺势疗法"[加权:扩展] OR "冥想运动疗法"[加权:扩展] OR "太极"[常用字段:智能] OR "八段锦"[常用字段:智能] OR "功法"[常用字段:智能] | 4928 |
| #10 | "灸法"[加权:扩展] OR "艾灸"[常用字段:智能] OR "隔物灸"[常用字段:智能] OR "艾条灸"[常用字段:智能] OR "雀啄灸"[常用字段:智能] OR "回旋灸"[常用字段:智能] OR "麦粒灸"[常用字段:智能] OR "瘢痕灸"[常用字段:智能] OR "悬起灸"[常用字段:智能] OR "艾炷灸"[常用字段:智能] OR "热敏灸"[常用字段:智能] | 20314 |
| #11 | "音乐疗法"[加权:扩展] OR "音乐"[常用字段:智能] | 8457 |
| #12 | "瑜伽"[加权:扩展] OR "舒展运动"[常用字段:智能] OR "放松"[常用字段:智能] | 7096 |
| #13 | "随机对照试验"[加权:扩展] OR "随机"[常用字段:智能] OR "随机对照"[常用字段:智能] OR "RCT"[常用字段:智能] | 1895053 |
| #14 | #2 OR #3 OR #4 OR #5 OR #6 OR #7 OR #8 OR #9 OR #10 OR #11 OR #12 OR #13 | 1440074 |
| #15 | #1 AND #13 AND #14 | 2785 |

**Supplementary Table S9.** Risk of bias assessment using ROB 2.0 tool

| **Study** | **Randomization process** | **Deviations from intended interventions** | **Missing outcome data** | **Outcome measurement** | **Selection of reported result** | **Overall Bias** |
|---|---|---|---|---|---|---|
| Cao-Y 2020 | Some concerns | Low | Low | Low | Low | Some concerns |
| Gao-SF 2016 | Some concerns | Low | Low | Some concerns | Low | Some concerns |
| Tang-HL 2015 | Some concerns | Low | Low | Low | Low | Some concerns |
| Li-Yj 2019 | Some concerns | Low | Low | Low | Low | Some concerns |
| Wang-N 2024 | Low | Low | Low | Low | Low | Low |
| Zhang-HJ 2010 | Some concerns | Low | Low | Low | Low | Some concerns |
| Zhao-J 2020 | Some concerns | Low | Low | Low | Low | Some concerns |
| Lin-JZ 2012 | Some concerns | Low | Low | Low | Low | Some concerns |
| Xuan-YB 2007 | Low | Low | Low | Low | Low | Low |
| Li-L 2019 | Some concerns | Low | Low | Low | Low | Some concerns |
| Wang-JP 2015 | Some concerns | Low | Low | Low | Low | Some concerns |
| Zhang-JX 2015 | Some concerns | Low | Low | Low | Low | Some concerns |
| Feng-XJ 2017 | Some concerns | Low | Low | Low | Low | Some concerns |
| Tu-JH 2012 | Low | Low | Low | Low | Low | Low |
| Xu-P 2015 | Low | Low | Low | Low | Low | Low |
| Jespersen-K 2019 | Some concerns | Low | Low | Low | Low | Some concerns |
| Jacobs-GD 2004 | Low | Low | Low | Low | Low | Low |
| Edinger-JD 2001 | Some concerns | Low | Low | Low | Low | Some concerns |
| Liang-XM 2017 | Low | Low | Low | Low | Low | Low |
| Yu-XP 2019 | Low | Low | Low | Low | Low | Low |
| Siu-PM 2021 | Some concerns | Low | Low | Low | Low | Some concerns |
| Christina-S 2007 | Some concerns | Low | Low | Low | Low | Some concerns |

| | | | | | | |
|---|---|---|---|---|---|---|
| Yin-X 2017 | Some concerns | Low | Low | Low | Low | Some concerns |
| Lee-B 2020 | Low | Low | Low | Low | Low | Low |
| Edinger-JD 2009 | Some concerns | Low | Low | Low | Low | Some concerns |
| Ritterband-LM 2009 | Low | Low | Low | Low | Low | Low |
| Yeung-WF 2009 | Some concerns | Low | Low | Low | Low | Some concerns |
| Van -SA 2014 | Low | Low | Low | Low | Low | Low |
| Scharf-MB 2007 | Some concerns | Low | Low | Low | Low | Some concerns |
| Feng-F 2019 | Some concerns | Low | Low | Low | Low | Some concerns |
| Espie-CA 2012 | Low | Low | Low | Low | Low | Low |
| Jernelov-S 2012 | Some concerns | Low | Low | Low | Low | Some concerns |
| Yeung-WF 2021 | Some concerns | Low | Low | Low | Low | Some concerns |
| Kaldo-V 2015 | Some concerns | Low | Low | Low | Low | Some concerns |
| Lichstein-KL 2001 | Some concerns | Low | Low | Low | Low | Some concerns |
| Khalsa-SBS 2021 | Low | Low | Low | Low | Low | Low |
| Riemann-D 2002 | Low | Low | Low | Low | Low | Low |
| Lovato-N 2014 | Low | Low | Low | Low | Low | Low |
| Passos-GS 2010 | Some concerns | Low | Low | Low | Low | Some concerns |
| Lo-C 2013 | Some concerns | Low | Low | Low | Low | Some concerns |
| Morin-CM 1999 | Low | Some concerns | Low | Low | Low | Some concerns |
| WU-R 2006 | Some concerns | Low | Low | Low | Low | Some concerns |
| Fu-C 2017 | Some concerns | Low | Low | Low | Low | Some concerns |
| Espie-CA 2007 | Some concerns | Low | Low | Low | Low | Some concerns |
| Abedian-Z 2015 | Low | Low | Low | Low | Low | Low |
| Huang-ZY | Low | Low | Low | Low | Low | Some concerns |

| | | | | | | |
|---|---|---|---|---|---|---|
| 2018 | | | | | | |
| Sivertsen-B 2006 | Some concerns | Low | Low | Low | Low | Some concerns |
| Jiang-L 2020 | Some concerns | Some concerns | Low | Low | Low | Some concerns |
| Wei-M 2013 | Some concerns | Low | Low | Low | Low | Some concerns |
| Wei-DM 2022 | Some concerns | Low | Low | Low | Low | Some concerns |
| Yang-HR 2024 | Low | Low | Low | Low | Low | Low |
| Zhuang-QX 2020 | Some concerns | Low | Low | Low | Low | Some concerns |
| Wei-M 2017 | Some concerns | Low | Low | Low | Low | Some concerns |

**Supplementary Figure S1.** Results of pairwise comparison on PSQI

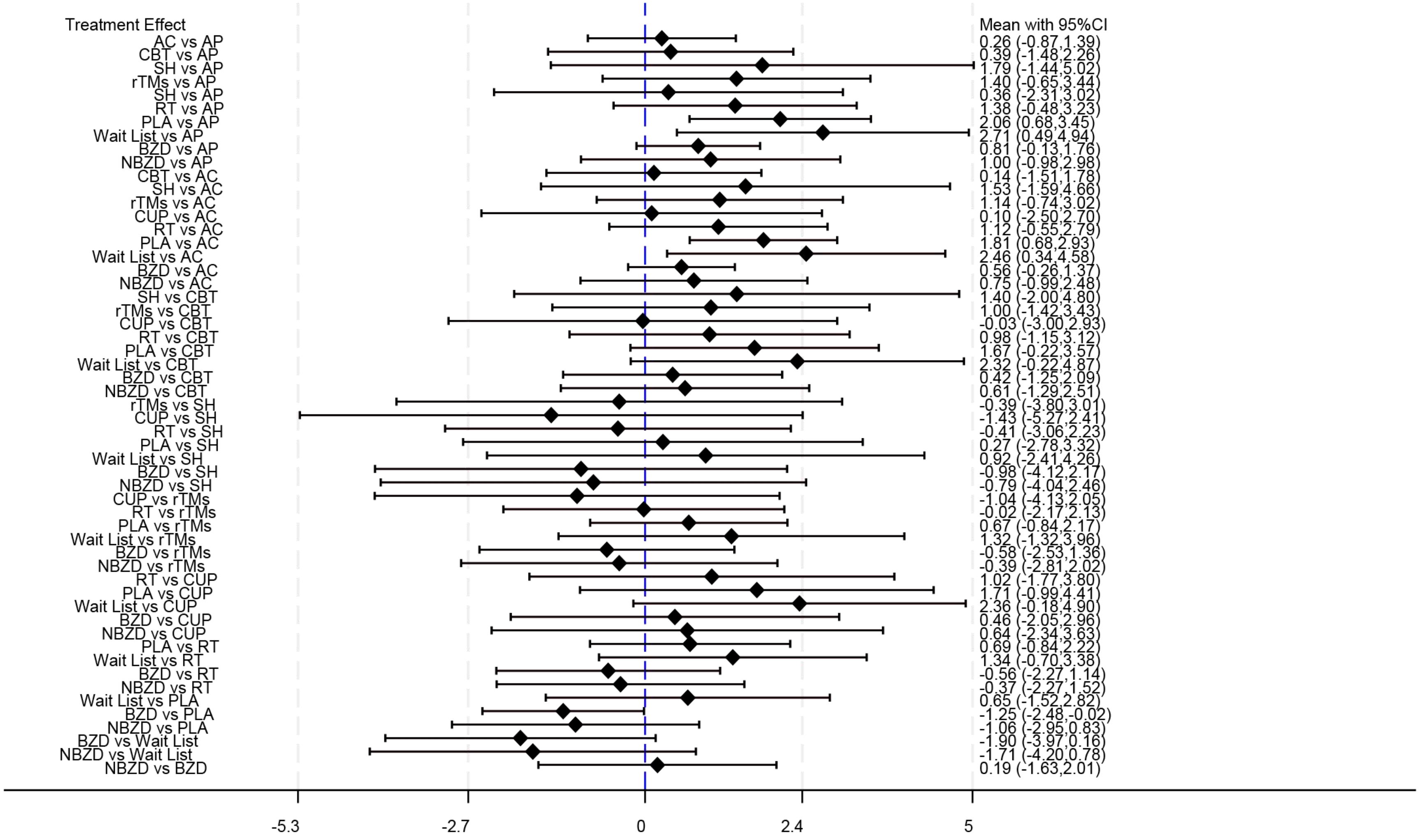

**Supplementary Figure S2.** Results of pairwise comparison on TST

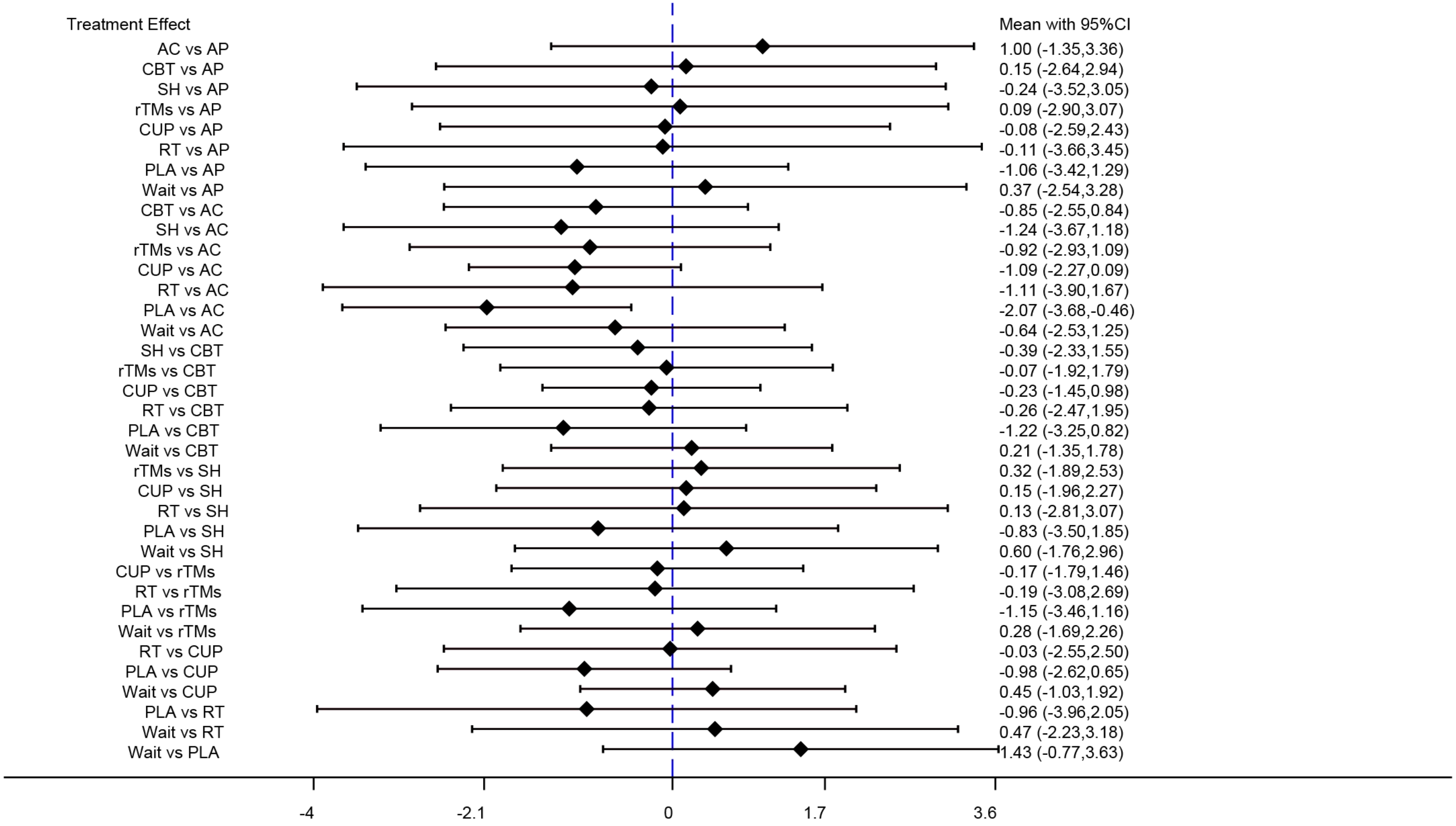

**Supplementary Figure S3.** Results of pairwise comparison on SE

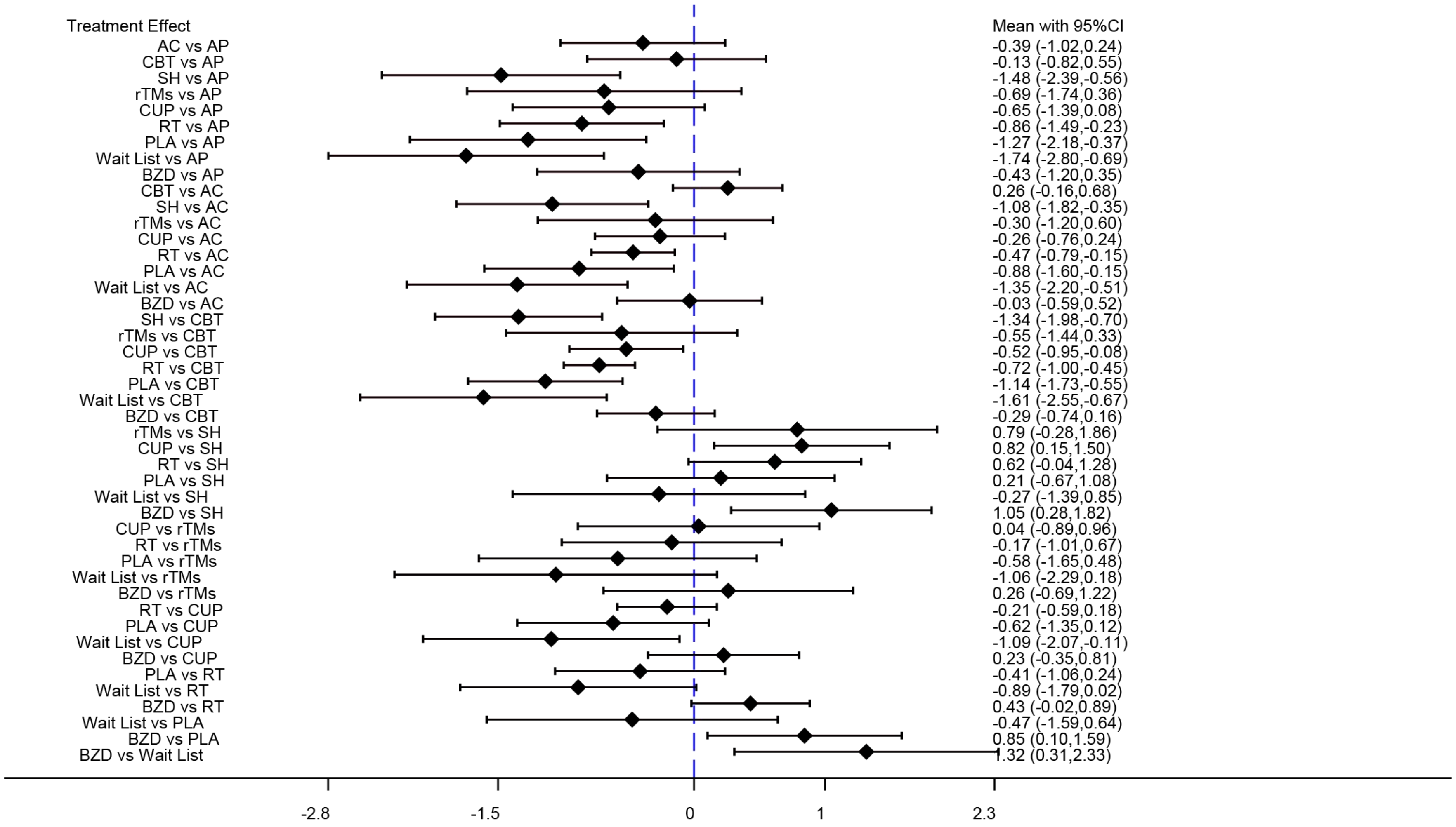

**Supplementary Figure S4.** Results of pairwise comparison on SL

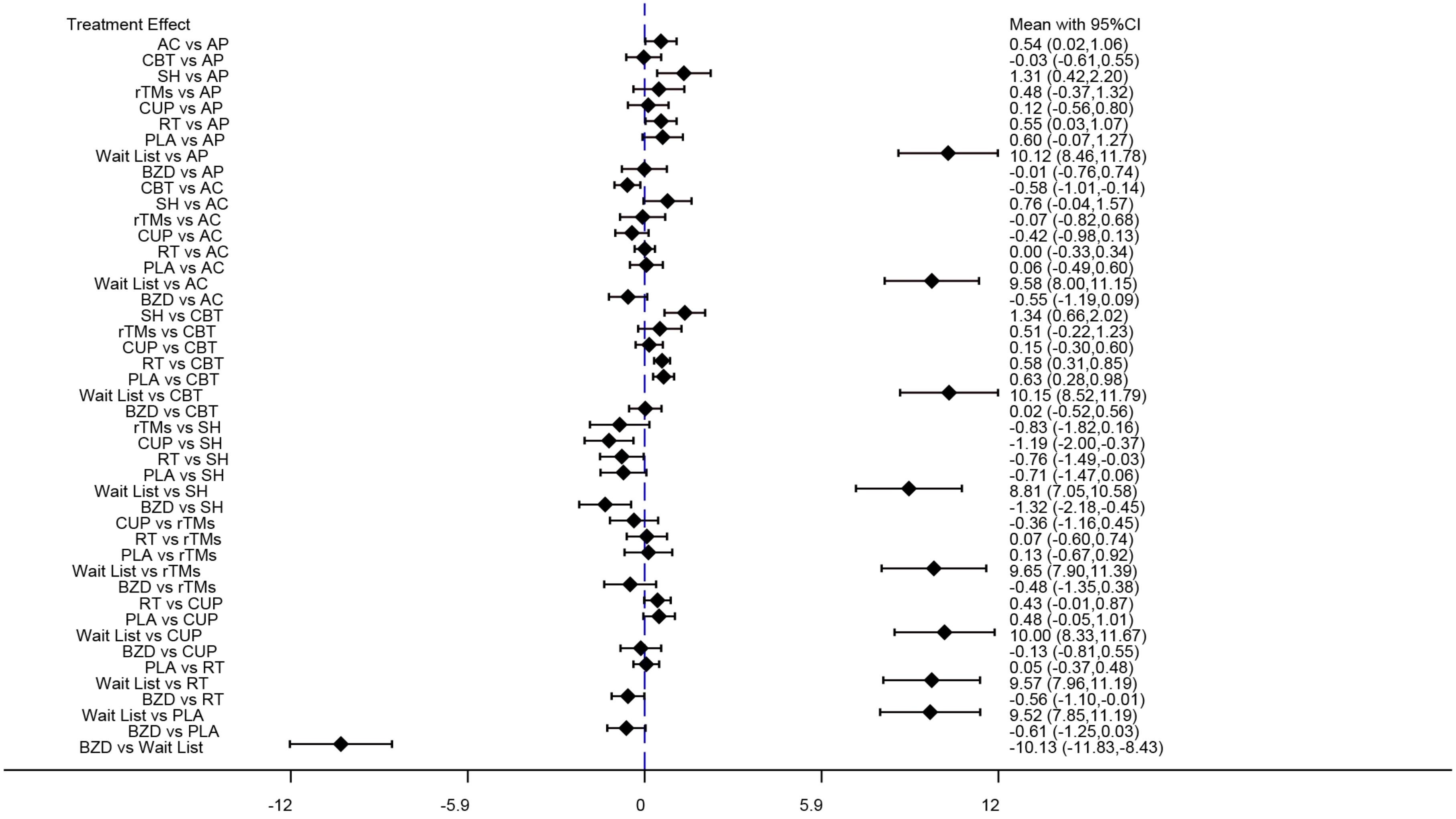

**Supplementary Figure S5.** Funnel plots for PSQI, TST, SE, and SL

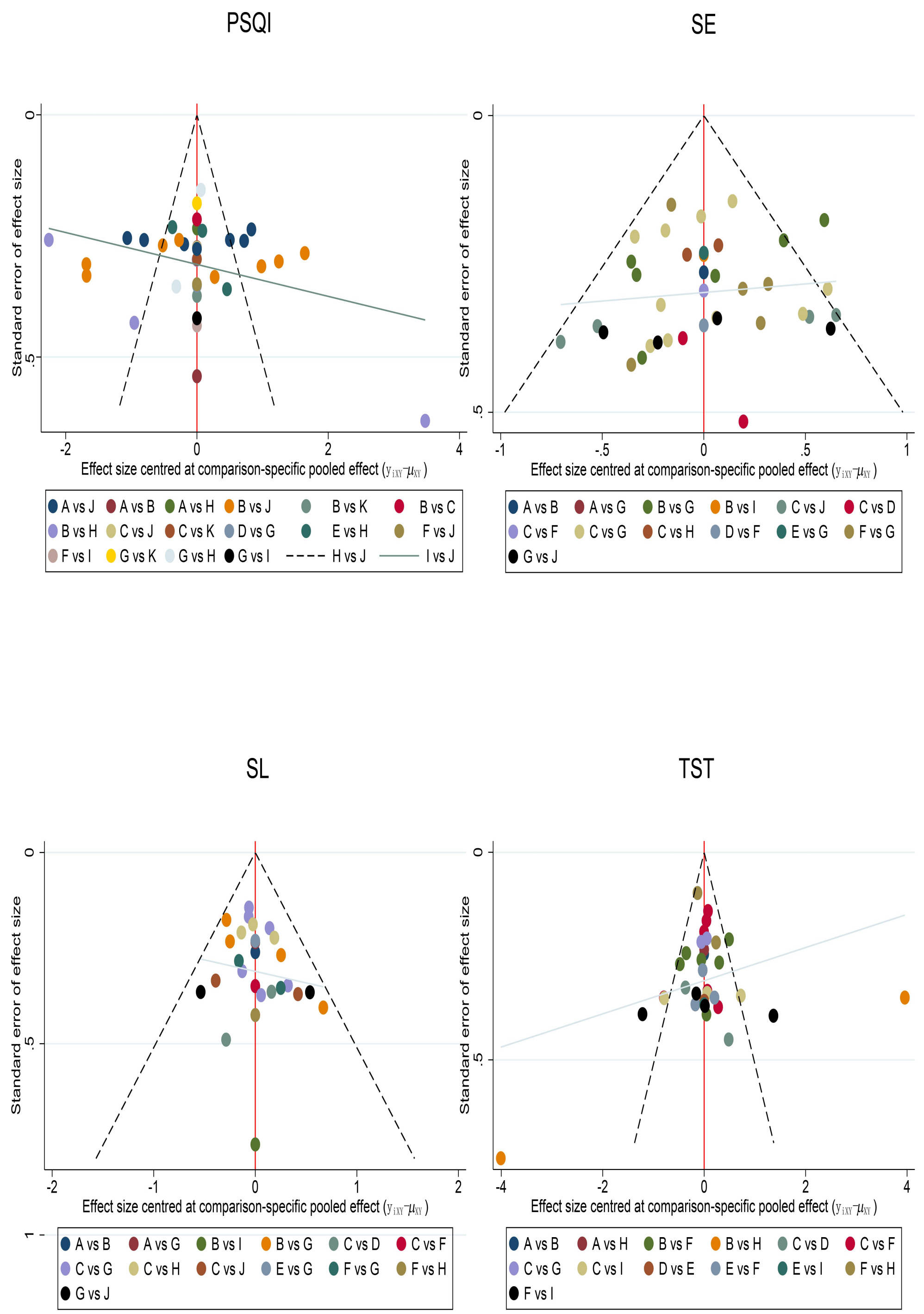


**Notes**: PSQI, Pittsburgh Sleep Quality Index; TST, Total Sleep Time; SE, sleep efficiency; SL, sleep latency; A, Acupressure/Massage (AP); B, Acupuncture (AC); C, Cognitive Behavioral Therapy (CBT); D, Sleep Hygiene (SH); E, Repetitive Transcranial Magnetic Stimulation (rTMS); F, Cupping Therapy (CUP); G, Relaxation Therapy (RT); H, Placebo (sham acupuncture or sham treatment) (PLA); I, Wait List; J, Benzodiazepines (BZD); K, Non-Benzodiazepines (NBZD).

**Supplementary Figure S6.** Average SUCRA values for PSQI

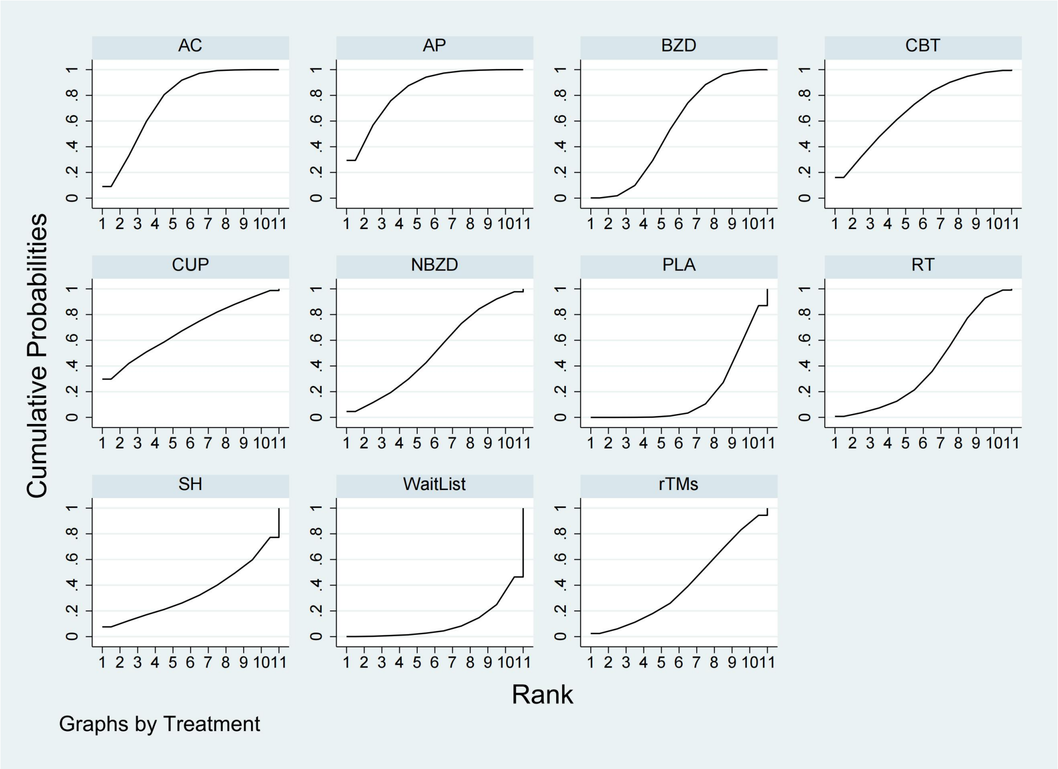


**Supplementary Figure S7.** Average SUCRA values for TST

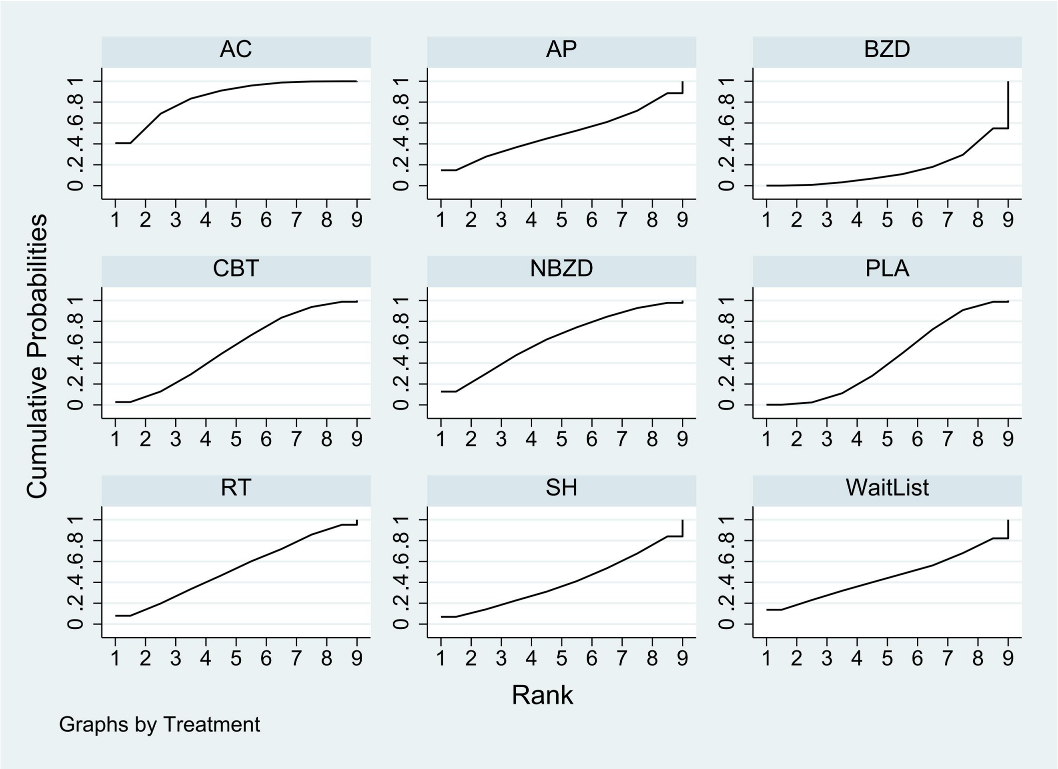

**Supplementary Figure S8.** Average SUCRA values for SE

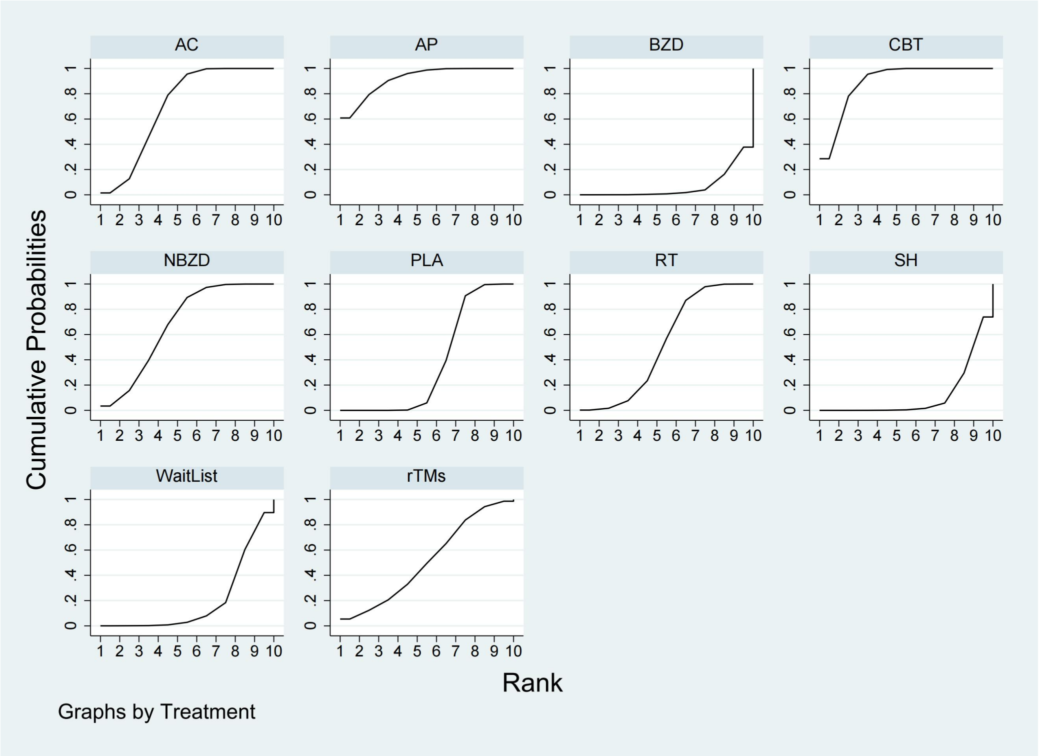


**Supplementary Figure S9.** Average SUCRA values for SL

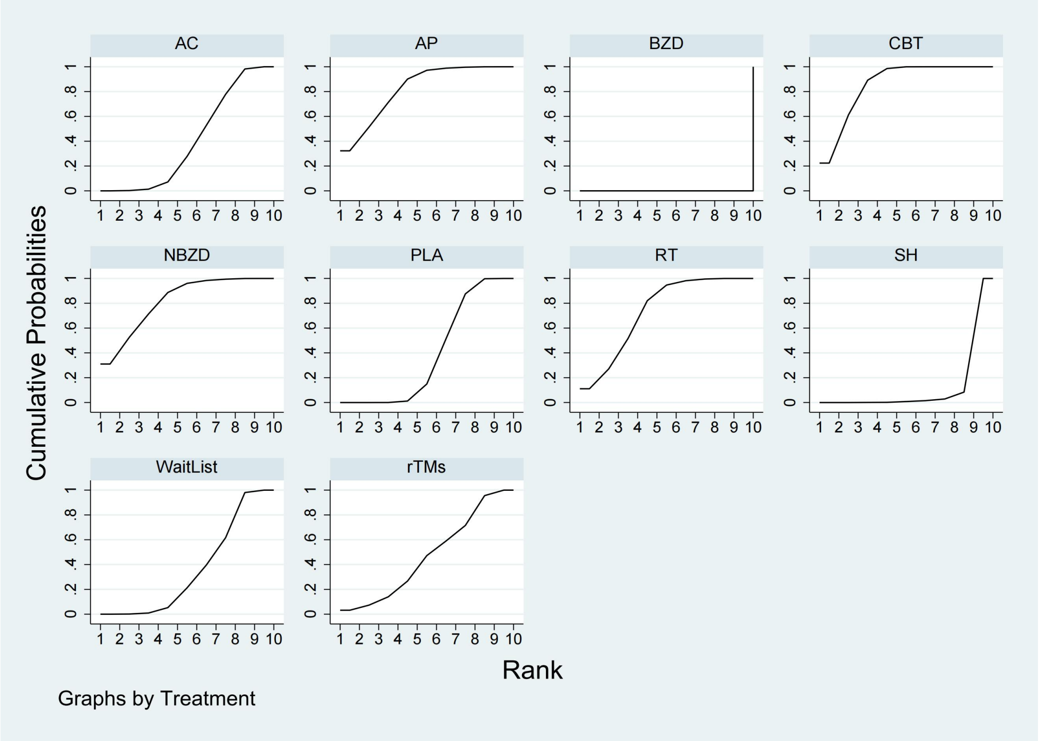


**Figures S6-9 Notes**: PSQI, Pittsburgh Sleep Quality Index; AC, Acupuncture; CBT, Cognitive Behavioral Therapy; AP, Acupressure/Massage; RT, Relaxation Therapy; CUP, Cupping Therapy; SH, Sleep Hygiene; rTMS, Repetitive Transcranial Magnetic Stimulation; BZD, Benzodiazepines; NBZD, Non-Benzodiazepines; PLA, Placebo (sham acupuncture or sham treatment); Wait List, Waiting List.

**Supplementary Figure S10.** Sensitivity analysis results

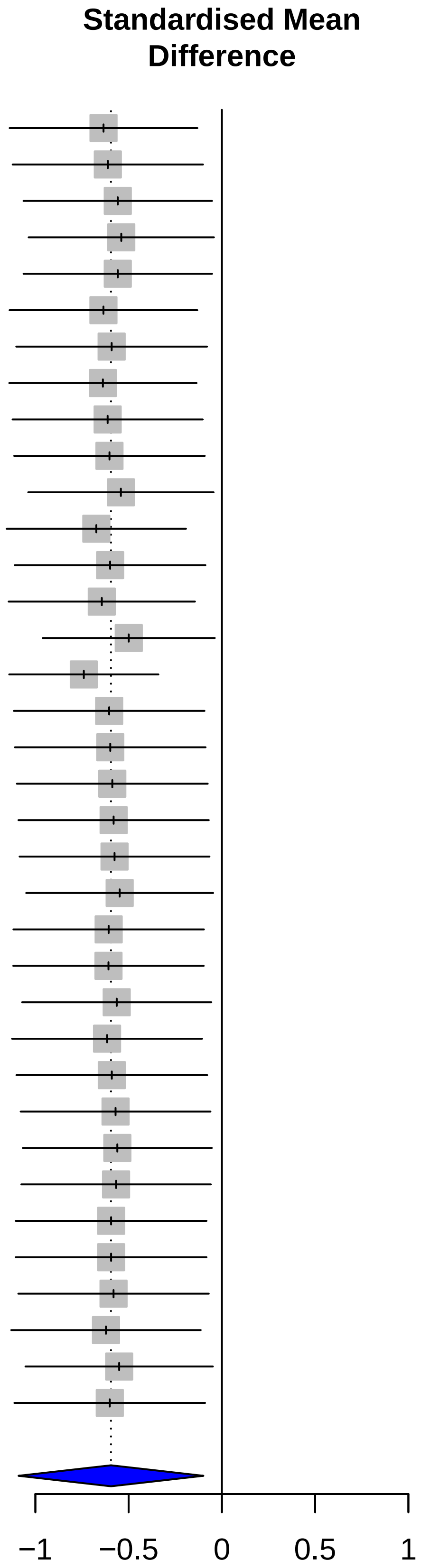


| Study | SMD | 95%−CI | P−value | Tau2 | Tau | I2 |
|---|---|---|---|---|---|---|
| Omitting Gao−SF_2016 1 | −0.63 | [−1.14; −0.13] | 0.0135 | 2.2003 | 1.4833 | 92.9% |
| Omitting Jiang−L_2020 2 | −0.61 | [−1.12; −0.10] | 0.0188 | 2.2610 | 1.5037 | 93.0% |
| Omitting Li−Yj_2019 3 | −0.56 | [−1.06; −0.05] | 0.0303 | 2.2167 | 1.4889 | 93.0% |
| Omitting Zhao−J_2020 4 | −0.54 | [−1.04; −0.04] | 0.0334 | 2.1399 | 1.4628 | 92.6% |
| Omitting Zhang−HJ_2010 5 | −0.56 | [−1.06; −0.05] | 0.0304 | 2.2167 | 1.4889 | 93.0% |
| Omitting Xuan−YB_2007 6 | −0.64 | [−1.14; −0.13] | 0.0134 | 2.1995 | 1.4831 | 92.9% |
| Omitting Feng−XJ_2017 7 | −0.59 | [−1.10; −0.08] | 0.0235 | 2.2736 | 1.5079 | 93.2% |
| Omitting Tu−JH_2012 8 | −0.64 | [−1.14; −0.14] | 0.0127 | 2.1877 | 1.4791 | 93.0% |
| Omitting Yeung−WF_2009 9 | −0.61 | [−1.12; −0.10] | 0.0185 | 2.2582 | 1.5027 | 93.1% |
| Omitting Feng−F_2019 10 | −0.60 | [−1.11; −0.09] | 0.0207 | 2.2682 | 1.5060 | 93.2% |
| Omitting Feng−F_2019 10 | −0.54 | [−1.04; −0.04] | 0.0327 | 2.1448 | 1.4645 | 93.0% |
| Omitting Feng−F_2019 10 | −0.67 | [−1.15; −0.19] | 0.0061 | 2.0021 | 1.4149 | 92.6% |
| Omitting Khalsa−SBS_2021 11 | −0.60 | [−1.11; −0.09] | 0.0215 | 2.2703 | 1.5068 | 93.2% |
| Omitting Li−L_2019 12 | −0.64 | [−1.14; −0.14] | 0.0115 | 2.1647 | 1.4713 | 92.8% |
| Omitting Li−L_2019 12 | −0.50 | [−0.96; −0.04] | 0.0339 | 1.8372 | 1.3554 | 92.6% |
| Omitting Li−L_2019 12 | −0.74 | [−1.14; −0.34] | 0.0003 | 1.3603 | 1.1663 | 92.0% |
| Omitting Zhang−YP_2018 13 | −0.60 | [−1.12; −0.09] | 0.0205 | 2.2691 | 1.5063 | 93.2% |
| Omitting Jespersen−KV_2019 14 | −0.60 | [−1.11; −0.09] | 0.0217 | 2.2706 | 1.5069 | 93.2% |
| Omitting Siu−PM_2021 15 | −0.59 | [−1.10; −0.08] | 0.0244 | 2.2735 | 1.5078 | 93.2% |
| Omitting Huang−ZY_2018 16 | −0.58 | [−1.09; −0.07] | 0.0258 | 2.2639 | 1.5046 | 93.2% |
| Omitting Lo−C_2013 17 | −0.58 | [−1.08; −0.07] | 0.0267 | 2.2553 | 1.5018 | 93.2% |
| Omitting Fu−C_2017 18 | −0.55 | [−1.05; −0.05] | 0.0321 | 2.1791 | 1.4762 | 92.8% |
| Omitting Abedian−Z_2015 19 | −0.61 | [−1.12; −0.10] | 0.0198 | 2.2662 | 1.5054 | 93.1% |
| Omitting Wei−M_2017 20 | −0.61 | [−1.12; −0.10] | 0.0196 | 2.2647 | 1.5049 | 93.2% |
| Omitting Wei−DM_2022 21 | −0.56 | [−1.07; −0.06] | 0.0293 | 2.2343 | 1.4947 | 93.0% |
| Omitting Yang−HR_2024 22 | −0.62 | [−1.12; −0.11] | 0.0178 | 2.2530 | 1.5010 | 93.1% |
| Omitting Wei−M_2013 23 | −0.59 | [−1.10; −0.08] | 0.0237 | 2.2729 | 1.5076 | 93.2% |
| Omitting Tang−HL_2015 24 | −0.57 | [−1.08; −0.06] | 0.0281 | 2.2491 | 1.4997 | 93.1% |
| Omitting Zhuang−QX_2020 25 | −0.56 | [−1.07; −0.05] | 0.0300 | 2.2252 | 1.4917 | 92.9% |
| Omitting Wang−N_2024 26 | −0.57 | [−1.08; −0.06] | 0.0287 | 2.2426 | 1.4975 | 93.1% |
| Omitting Lin−JZ_2012 27 | −0.59 | [−1.11; −0.08] | 0.0229 | 2.2743 | 1.5081 | 93.2% |
| Omitting Wang−JP_2015 28 | −0.59 | [−1.11; −0.08] | 0.0229 | 2.2736 | 1.5078 | 93.2% |
| Omitting Zhang−JX_2015 29 | −0.58 | [−1.09; −0.07] | 0.0258 | 2.2661 | 1.5054 | 93.2% |
| Omitting Xu−P_2015 30 | −0.62 | [−1.13; −0.11] | 0.0165 | 2.2403 | 1.4968 | 93.0% |
| Omitting Liang−XM_2017 31 | −0.55 | [−1.05; −0.05] | 0.0317 | 2.1901 | 1.4799 | 92.9% |
| Omitting Yu−XP_2019 32 | −0.60 | [−1.11; −0.09] | 0.0212 | 2.2710 | 1.5070 | 93.2% |
| **Random effects model** | **−0.59** | **[−1.09; −0.10]** | **0.0187** | **2.1923** | **1.4806** | **93.0%** |

**Figure S10 Notes**: Sensitivity analysis of standardized mean difference (SMD) using a random effects model. Each point represents the SMD for a specific study, with 95% CI error bars. Studies are listed on the y-axis, and SMD values are on the x-axis. The analysis omits one study at a time to assess the impact on overall results. SMD: effect size of the intervention. 95% CI: confidence interval for SMD. P-value: statistical significance. $Tau^2$: between-study variance. Tau: square root of $Tau^2$, indicating the standard deviation of the true effects across studies. $I^2$: heterogeneity among studies.

**Supplementary Figure S11.** Subgroup analysis based on treatment duration

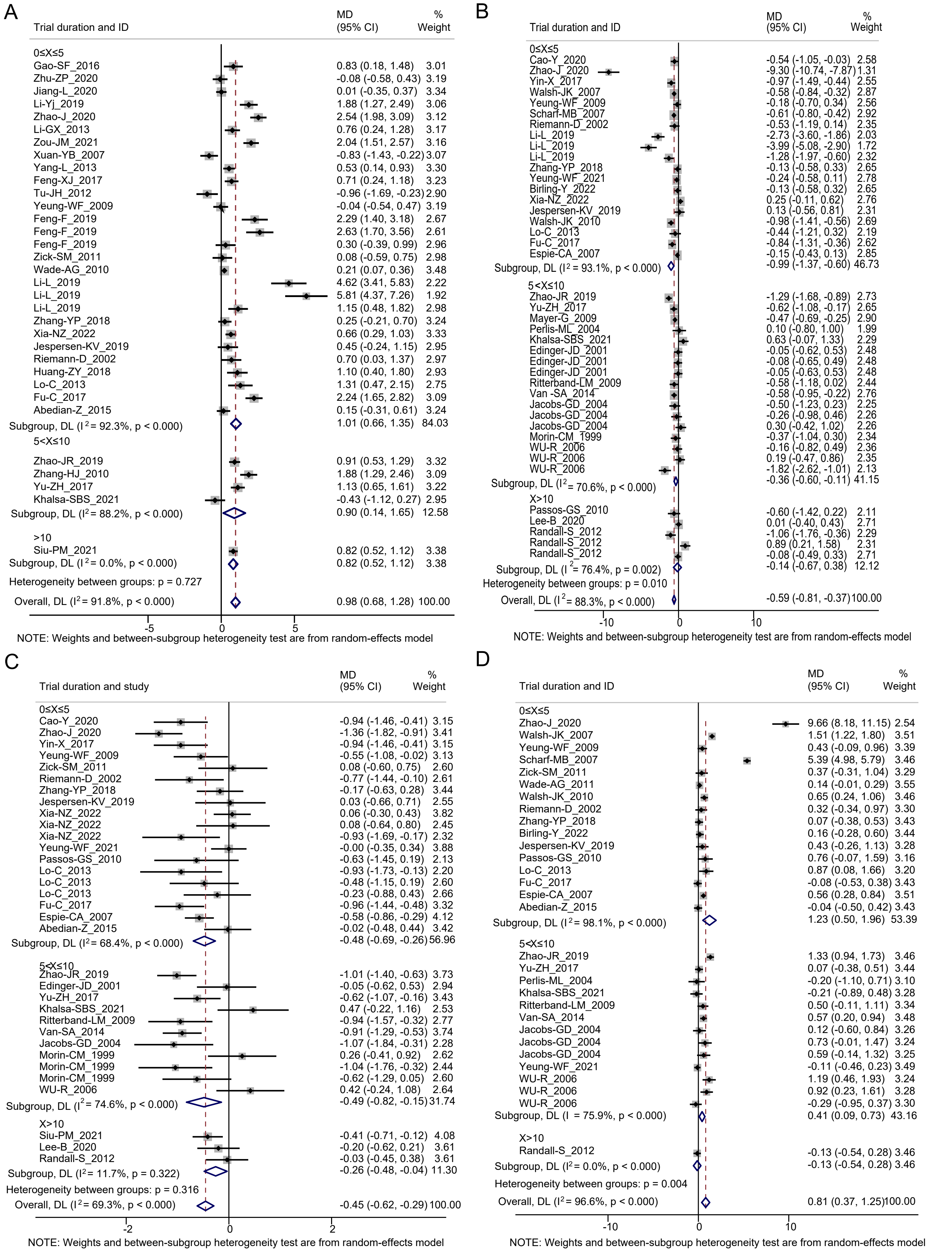


**Figures S11 Notes**: A: PSQI forest plot; B: TST forest plot; C: SE forest plot; D: SL forest plot. This forest plot illustrates the mean difference (MD) and 95% confidence intervals (CIs) for the PSQI, TST, SE and SL under different treatment duration (subgrouped by weeks). Each point represents the effect size of an individual study, with horizontal lines indicating their CIs.

**Supplementary Figure S12.** Subgroup analysis based on disease course

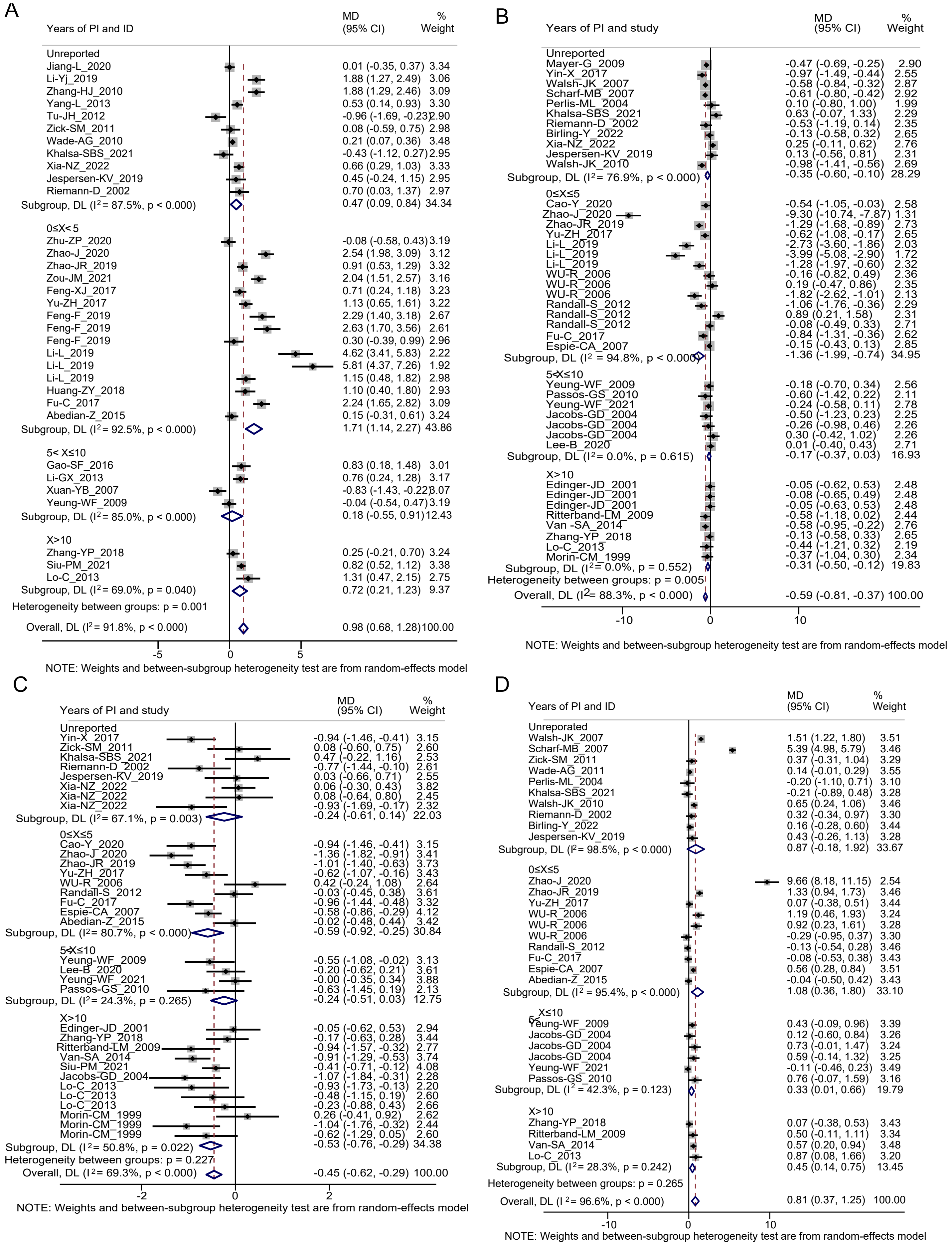


**Figures S12 Notes**: A: PSQI forest plot; B: TST forest plot; C: SE forest plot; D: SL forest plot. This forest plot illustrates the mean difference (MD) and 95% confidence intervals (CIs) for the PSQI, TST, SE and SL under different treatment duration (subgrouped by weeks). Each point represents the effect size of an individual study, with horizontal lines indicating their CIs.
